# Current Status of the Epidemiologic Evidence Linking Polychlorinated Biphenyls and Non-Hodgkin Lymphoma, and the Role of Immune Dysregulation

**DOI:** 10.1289/ehp.1104652

**Published:** 2012-05-02

**Authors:** Shira Kramer, Stephanie Moller Hikel, Kristen Adams, David Hinds, Katherine Moon

**Affiliations:** Epidemiology International, Hunt Valley, Maryland, USA

**Keywords:** cancer, immunologic response, non-Hodgkin lymphoma, organochlorines, polychlorinated biphenyls

## Abstract

Background: Although case–control studies conducted to date have largely affirmed the relationship between polychlorinated biphenyls (PCBs) and non-Hodgkin lymphoma (NHL), occupational cohort studies of PCB-exposed workers have been generally interpreted as negative, thereby raising doubts about a potential causal association. A common theme of immune dysregulation unifies many of NHL’s strongest risk factors, and several authors have posited that subclinical immune dysregulation may increase NHL risk by decreasing host resistance, reducing control of cellular proliferation and differentiation, and diminishing tumor surveillance mechanisms.

Objectives: The goals of this review were *a*) to evaluate the epidemiological research examining the association between PCB exposure and NHL and discuss the contribution to the weight of evidence of case–control studies and occupational cohort studies; and *b*) to summarize the evidence for immune dysregulation as a means by which PCBs may cause NHL.

Methods: We performed a literature search using PubMed and seven additional online biomedical and toxicological referencing libraries to identify literature published through August 2011.

Discussion and Conclusions: Overall, we conclude that the weight of evidence supports a causal role of PCBs in lymphomagenesis. The strongest epidemiological evidence for the relationship between PCBs and NHL comes from case–control studies conducted among the general population. Epidemiological and toxicological data demonstrating immunosuppressive and inflammatory effects of PCBs further contribute to the weight of evidence by providing a plausible explanation for how PCBs can cause NHL through immune dysregulation.

Non-Hodgkin lymphoma (NHL), a group of lymphoid malignancies that originate from B cells, T cells, or natural killer (NK) cells, is one of the most commonly diagnosed cancers in the United States and worldwide (Fisher and Fisher 2004). In the United States, the incidence of NHL grew precipitously from the 1950s to the 1990s, increasing by an average of 3.6% per year from 1975 through 1991, and continued to rise over the period 1991–2008, albeit at a slower rate of 0.5% per year on average (Devesa and Fears 1992; Howlader et al. 2011).

Acquired and congenital immune deficiencies greatly increase the risk of developing NHL; however, these conditions are relatively uncommon in the population and cannot explain the drastic increases in NHL incidence (Grulich et al. 2007; Hartge and Devesa 1992). Changes in cancer reporting, diagnosis, and classification systems for lymphomas are also not sufficient to explain these trends (Banks 1992; Hartge and Devesa 1992). A recent analysis of age-period-cohort models for NHL incidence in the Doubs region of France from 1980 through 2005 identified a strong period effect in increased NHL incidence in the years 1983–1992 across all age groups, which the authors determined to be consistent with one or more ubiquitous environmental exposures emerging during the 1960s (Viel et al. 2010).

Polychlorinated biphenyls (PCBs) were first manufactured in large scale in the 1920s and reached peak production by the 1960s, consistent with the period effect of an emerging environmental exposure observed by Viel et al. (2010). PCBs are chemically stable, lipid-soluble compounds that are resistant to degradation and thus accumulate in the environment, food chain, and human tissues. In 1979, the U.S. Environmental Protection Agency (EPA) banned further production of PCBs as a result of their environmental persistence and the potential for adverse health effects (U.S. EPA 1979). However, exposure continues through consumption of fish and other food sources in which PCBs have bioaccumulated, exposure to water and soil containing PCBs that have not degraded, and inhalation of PCBs volatilized from products manufactured before the production ban.

Initial epidemiological studies of the link between PCBs and NHL (Hardell et al. 1996, 1997; Rothman et al. 1997) were prompted by the convergence of several important factors: the temporal correspondence between exposure to PCBs and increasing incidence of NHL; the toxicological and epidemiological evidence for the immunotoxicity and carcinogenicity of PCBs; and the structural similarity between PCBs and dioxins, which are known human carcinogens that have also been associated with NHL. Since the publication of these initial studies in the late 1990s, > 15 case–control studies have assessed the relationship between PCB exposure and NHL in the general population, and most (e.g., Bertrand et al. 2010; De Roos et al. 2005; Rothman et al. 1997; Spinelli et al. 2007; Viel et al. 2011) but not all (Cocco et al. 2008; Laden et al. 2010; Nordstrom et al. 2000) have found positive associations between NHL and PCBs sampled from biological tissues or carpet dust. In contrast, cohort studies of PCB-exposed workers have been generally interpreted as negative, thereby creating uncertainty about a potential causal association.

An updated assessment of the weight of evidence is warranted because several case–control studies have been published since the most recent reviews of PCBs and NHL (Engel et al. 2007b; Golden and Kimbrough 2009). Furthermore, in evaluating the evidence regarding causation, it is important to examine the methodological characteristics of the case–control and occupational cohort literature to assess their relative contributions to the weight of evidence.

## Methods

We framed our literature search and analysis around two questions: *a*) What is the weight of evidence for a causal association between human exposure to PCBs and NHL? And *b*) do the immunomodulatory effects of PCBs provide a plausible explanation for the positive PCB–NHL associations observed in the epidemiological case–control literature?

*Question 1: PCB–NHL weight of evidence analysis.* For this review, we defined a weight of evidence analysis as the formal qualitative review and synthesis of a body of literature, which may include data from numerous disciplines, including human epidemiological, toxicological, and mechanistic studies. In our weight of evidence analysis, we focused on the epidemiological literature examining the relationship between PCB exposure and NHL. Judgments were made regarding the potential for bias, internal and external validity, strength and consistency of results, and the contribution of each study to the overall weight of evidence. Conclusions were based on the convergence of the evidence (Krimsky 2005).

To develop a database of literature to be considered, we performed searches from 1980 through August 2011 in PubMed (http://www.ncbi.nlm.nih.gov/pubmed), Embase (http://www.embase.com), Scopus (http://www.scopus.com), TOXNET (http://toxnet.nlm.nih.gov), CINAHL (http://www.ebscohost.com/cinahl), Scirus (http://www.scirus.com), BioMed Central (http://www.biomedcentral.com), and ISI Web of Knowledge (http://www.webofknowledge.com) [see Supplemental Material, p. 3 for key words used in the search (http://dx.doi.org/10.1289/ehp.1104652)]. We reviewed the reference lists of key articles to ensure adequacy and completeness of the literature database, and limited article retrieval to articles written in English or with English translations available.

Studies included in the weight of evidence analysis were required to meet both of the following criteria: *a*) measurement or characterization of exposure to PCBs or industrial mixtures containing PCBs; and *b*) examination of NHL or lymphoma in humans as an outcome. A total of four conference abstracts were excluded because they reported data subsequently published elsewhere in manuscript form, and five studies were excluded because authors performed no statistical analyses of the main effect of PCBs on NHL/lymphoma or reported *p*-values but failed to report magnitudes of effect [for number of citations retrieved and excluded, see Supplemental Material, p. 5 (http://dx.doi.org/10.1289/ehp.1104652)]. We relied on the statistical calculations performed by study authors, and did not perform any post hoc analyses. In all studies, alpha was set by the study authors at 0.05.

Our weight of evidence analysis included a total of 19 case–control studies (see Supplemental Material, Table S1 (http://dx.doi.org/10.1289/ehp.1104652)] and 17 cohort studies (see Supplemental Material, Table S2). We considered all studies meeting inclusion criteria, regardless of the results or conclusions of study authors. In summarizing the weight of evidence in this review, we emphasized studies that we considered contributed most strongly to the weight of evidence; however, data from all 36 studies are presented in the Supplemental Material (see Supplemental Material, Tables S1 and S2).

*Question 2: evidence for the role of immune dysregulation in linking PCB exposure and NHL risk.* The immunomodulatory effects of PCBs and dioxins in animals and humans have been extensively reviewed and summarized elsewhere [Agency for Toxic Substances and Disease Registry (ATSDR) 2000; Kerkvliet 2002; Levin et al. 2005; Tryphonas 1995; Yoshizawa et al. 2007], and immune dysregulation is an important and well-established risk factor for NHL (e.g., Grulich et al. 2007). Building on the evidence provided by these and other authors, we performed a literature search using PubMed, Embase, Scopus, TOXNET, and Scirus to identify any direct or indirect evidence linking PCB exposure to NHL through immune effects [for key words used in the search, see Supplemental Material, p. 3 (http://dx.doi.org/10.1289/ehp.1104652)].

The epidemiological literature regarding the immunotoxicity of environmental contaminants such as PCBs is complex and continues to evolve (Selgrade 1999, 2007), and we did not determine a weight of evidence analysis to be possible because of the limited quantity of studies examining any single marker or end point and the continued uncertainties regarding the relevance of certain cellular-level immune effects to population-level disease risks (Selgrade 1999). However, review of the resultant epidemiological and toxicological literature suggested several indirect lines of evidence supporting the hypothesis that the effects of PCBs on the immune system provide a plausible explanation for the positive PCB–NHL associations observed in the epidemiological literature. Studies in support of this hypothesis are cited and discussed in the main text of this review.

## Epidemiological Studies of NHL Risks Associated with PCB Exposure

*Case–control studies.* Case–control studies of PCB exposure and NHL have estimated general population exposure to environmental and dietary sources of PCBs by measuring PCB levels in either biological sources (serum, plasma, adipose tissue) or nonbiological sources (carpet dust, soil). These studies measured levels of multiple PCB congeners and presented results for total PCBs, individual congeners, and/or various congener groupings such as dioxin-like PCBs (DL-PCBs) [PCBs that act through a mechanism of toxicity similar to that of dioxins (Van den Berg et al. 1998)], non-dioxin-like PCBs (NDL-PCBs) (PCBs not classified as DL-PCBs), moderately and highly chlorinated PCBs [based on the number of chlorine atoms attached to the biphenyl molecule (Moysich et al. 1999)], and immunotoxic PCBs [based either on the PCB classification system proposed by Wolff et al. (1997) or on epidemiological studies of PCBs and immune parameters (Daniel et al. 2001; Van Den Heuvel et al. 2002)]. In general, exposures were categorized based on the distribution of PCB levels among controls, and odds ratios (ORs) for NHL were calculated by comparing higher exposure quantiles to the lowest referent quantile.

Several nested case–control studies were conducted within four prospective cohorts: the Campaign Against Cancer and Stroke (CLUE I) in Washington County, Maryland (Engel et al. 2007a; Rothman et al. 1997); the U.S.-based Nurses’ Health Study (NHS) (Engel et al. 2007a; Laden et al. 2010) and Physicians’ Health Study (PHS) (Bertrand et al. 2010); and the Norwegian Janus cohort (Engel et al. 2007a) (Table 1). The earliest study (Rothman et al. 1997), nested within the 1974 CLUE I cohort, analyzed serum samples collected an average of 12.1 years before case diagnosis from 74 NHL cases and 147 controls. This study found a significant dose–response trend for total serum PCBs and NHL (*p* = 0.0008), and reported a statistically significant OR of 4.5 [95% confidence interval (CI): 1.7, 12.0] for the highest versus lowest exposure group. In a subsequent analysis of the CLUE I cohort that estimated congener-specific risks among the same 74 cases and 147 controls (Engel et al. 2007a), results mirrored those observed in the Rothman analysis, with significant dose–response trends observed for total PCBs and for PCBs 118, 138, and 153 [Table 1; see also Supplemental Material, Table S1 (http://dx.doi.org/10.1289/ehp.1104652)]. Engel et al. (2007a) presented similar analyses conducted within the Janus cohort, using serum samples from 190 case–control pairs collected 16.6 years (median) before case diagnosis, and also found NHL to be positively associated with total PCBs and PCBs 118, 138, and 153 (OR range: 1.7–2.0 in the highest exposure quartiles) (Table 1).

**Table 1:** Selected findings^a^ from prospective nested case–control studies examining levels of PCBs in blood and risk of NHL

Reference	Cohort	Cases/controls^b^	Exposure	Highest versus lowest quantile OR (95% CI)^c^	p for trend^d^
Rothman et al. 1997		CLUE I		74/147		ΣPCBs		4.5 (1.7, 12.0)		0.0008
Engel et al. 2007a		CLUE I		74/147		ΣPCBs		4.6 (1.7, 12.7)		< 0.005
						PCB-118		5.4 (1.7, 17.1)		< 0.05
						PCB-138		4.4 (1.5, 12.6)		< 0.05
						PCB-153		2.2 (0.9, 5.2)		< 0.05
		Janus		190/190		ΣPCBs		1.7 (0.8, 3.4)		NS
						PCB-118		1.7 (0.9, 3.5)		< 0.05
						PCB-138		1.7 (0.8, 3.2)		< 0.05
						PCB-153		2.0 (1.0, 3.9)		< 0.05
		NHS		30/78		ΣPCBs		4.7 (1.2, 18.9)		0.012
						PCB-118		3.3 (0.9, 12.4)		0.022
						PCB-138		3.8 (1.1, 13.6)		0.026
						PCB-153		3.2 (0.9, 11.8)		NS
Laden et al. 2010		NHS		145/290		ΣPCBs		1.02 (0.53, 1.95)		NS
						ΣImmunotoxic PCBse		0.89 (0.45, 1.77)		NS
						Σ(118,138,153,180)		0.91 (0.48, 1.75)		NS
						PCB-118		0.81 (0.42, 1.56)		NS
						PCB-138		0.95 (0.49, 1.83)		NS
						PCB-153		0.82 (0.43, 1.56)		NS
						PCB-180		1.03 (0.52, 2.02)		NS
Bertrand et al. 2010		PHS		205/409		ΣPCBs		1.6 (0.91, 2.9)		< 0.01
						ΣImmunotoxic PCBse		1.4 (0.8, 2.6)		NS
						Σ(118,138,153,180)		1.8 (1.0, 3.2)		< 0.01
						PCB-118		1.4 (0.76, 2.5)		NS
						PCB-138		1.8 (0.98, 3.2)		0.02
						PCB-153		2.1 (1.1, 3.8)		< 0.01
						PCB-180		2.4 (1.3, 4.5)		< 0.01
Abbreviations: CLUE I, Campaign Against Cancer and Stroke Study; NHS, Nurses’ Health Study; NS, nonsignificant; OR, odds ratio; PCB, polychlorinated biphenyl; PHS, Physicians’ Health Study; Σ, total. aOdds ratios for the highest versus lowest exposure quantile are shown. Additional findings are reported in Supplemental Material, Table S1 (http://dx.doi.org/10.1289/ehp.1104652). bSample sizes provided for total cohort; all controls are matched except Engel et al.’s (2007a) analysis of NHS cohort (see Supplemental Material, Table S1 for description of matching factors). cAdjusted odds ratios, except Rothman et al. (1997) (see Supplemental Material, Table S1 for description of adjustment factors); all odds ratios compare fourth versus first quartile, except Engel et al.’s (2007a) analysis of NHS cohort (third versus first tertile) and Bertrand et al.’s (2010) (fifth versus first quintile). dExact p-value given when possible, otherwise information given in the format provided by study authors. eAs defined by Wolff et al. (1997).										

An analysis of the NHS cohort by Engel and colleagues (2007a) based on 30 female NHL cases and 78 controls found that the odds of NHL were consistently elevated in the second and third versus first tertiles for total PCBs and PCBs 118, 138, and 153, with ORs ranging from 3.2–4.7 in the highest tertile (Table 1). However, this analysis had methodological limitations such as a small sample size and a median duration between blood sample collection and NHL diagnosis of only 1.04 years. In contrast, a 2010 analysis of this cohort with a larger sample size (145 cases and 290 controls), based on plasma collected 5.8 years (median) before diagnosis, and which adjusted for more potential confounders including timing of blood collection, region, and breast-feeding, did not observe associations between NHL and increasing quartiles of PCB exposure for any of the examined congeners or congener groups (Laden et al. 2010) (Table 1). Employing similar methodology within the PHS cohort and using plasma samples from 205 male NHL cases and 409 controls collected 12 years (median) before case diagnosis, Bertrand et al. (2010) found significant dose–response trends for total PCBs and PCBs 138, 153, and 180 (OR range: 1.4–2.4 for the highest vs. lowest exposure quintiles) (Table 1).

Consistent with these case–control studies nested within prospective cohorts, retrospective population-based case–control studies have also generally demonstrated positive associations between PCB exposure and NHL. In a case–control study that analyzed plasma samples from Surveillance, Epidemiology, and End Results (SEER) registry cases (*n* = 100) and population-based controls (*n* = 100), De Roos et al. (2005) observed positive dose–response trends for PCB-156 (*p* = 0.03 for trend, highest vs. lowest quartile OR = 2.7, 95% CI: 0.97, 7.5); PCB-180 (*p* = 0.01 for trend, highest vs. lowest quartile OR = 3.5, 95% CI: 1.3, 9.2); and PCB-194 (*p* = 0.04 for trend, highest vs. lowest quartile OR = 2.7, 95% CI: 1.04, 6.9). Examining PCB levels on a continuous scale found increased risks of NHL associated with each 10-ng/g lipid increase in levels of PCB-169 (OR = 1.47; 95% CI: 1.13, 1.91); PCB-194 (OR = 1.5; 95% CI: 0.98, 2.2); PCB-180 (OR = 1.1; 95% CI: 0.98, 1.2); PCB-170 (OR = 1.2; 95% CI: 0.94, 1.6); and PCB-156 (OR = 1.7; 95% CI: 0.91, 3.1). A series of analyses (Colt et al. 2005, 2009; Morton et al. 2008) confirmed that the associations observed between plasma PCBs and NHL risk in the De Roos et al. (2005) study population were also present for levels of PCBs measured in carpet dust [see Supplemental Material, Table S1 (http://dx.doi.org/10.1289/ehp.1104652)]. A large population-based case–control study in British Columbia with 422 NHL cases and 460 controls (Spinelli et al. 2007) found significant dose–response trends for > 10 congeners or congener groupings, including total PCBs (*p* = 0.001); total DL-PCBs (*p* < 0.001) and NDL-PCBs (*p* < 0.001); and PCBs 118 (*p* = 0.004), 153 (*p* = 0.002), and 180 (*p* = 0.005) (see Supplemental Material, Table S1).

Several case–control studies conducted in Sweden identified associations between NHL and PCB levels above versus below the median for total PCBs (Hardell et al. 2001, 2009), immunotoxic PCBs (Hardell et al. 2001, 2009), moderately chlorinated PCBs (Hardell et al. 2009), and highly chlorinated PCBs (Hardell et al. 2009). But a study restricted to cases with hairy cell leukemia, a rare subtype of NHL, did not find an association with PCBs (Nordstrom et al. 2000) [see Supplemental Material, Table S1 (http://dx.doi.org/10.1289/ehp.1104652)]. A multisite study in France, Germany, and Spain (Cocco et al. 2008) with 174 NHL cases and 203 controls did not observe an association between PCBs and NHL in the overall study population (see Supplemental Material, Table S1).

Two recent studies examined NHL risk in populations living near point sources of PCB exposure. A study of incident NHL cases (*n* = 34) and population-based controls (*n* = 34) from an area surrounding a municipal solid waste incinerator in Besançon, France, found increased odds of NHL associated with increasing serum levels of DL-PCBs (OR = 1.04; 95% CI: 1.00, 1.07); NDL-PCBs (OR = 1.02; 95% CI: 1.01, 1.05); the sum of polychlorinated dibenzodioxins (PCDDs), polychlorinated dibenzofurans (PCDFs) and DL-PCBs (OR=1.04; 95% CI: 1.01, 1.5); and 12 of the 14 measured PCB congeners (Viel et al. 2011). Similarly, a population-based case–control study with 495 NHL cases (287 incident cases and 208 deaths) and 1,467 controls conducted in an Italian city with considerable soil contamination from a PCB-production plant found that residence in a contaminated area (based on PCB levels in soil samples) was associated with elevated odds of incident NHL or NHL deaths (Maifredi et al. 2011) [see Supplemental Material, Table S1 (http://dx.doi.org/10.1289/ehp.1104652)].

Three additional case–control studies had mixed findings but ultimately did not contribute strongly to the weight of evidence because of a variety of methodological limitations (Fritschi et al. 2005; Greenland et al. 1994; Quintana et al. 2004) (see Supplemental Material, Table S1).

*Comments on case–control studies.* On the basis of our evaluation of all case–control studies that examined the association between PCB exposure and NHL [see Supplemental Material, Table S1 (http://dx.doi.org/10.1289/ehp.1104652)], we conclude that the preponderance of findings from case–control studies supports a causal relationship between exposure to PCBs and NHL in the general population. These associations have been observed across summary metrics of PCB exposure (e.g., total PCBs, immunotoxic PCBs) as well as with individual congeners. The actual PCB congeners identified in the case–control studies as associated with increased NHL risk represent “markers” for more general PCB exposure because of their biological prevalence, probability of detection in biological samples, and degree of correlation, and do not necessarily represent the full spectrum of PCB exposures or risks posed by environmental exposure to PCBs.

Potential confounding factors of the association between PCBs and NHL have not shown evidence of substantial confounding in the case–control studies, including concomitant exposure to dioxins, furans, and/or organochlorine pesticides (Bertrand et al. 2010; Cocco et al. 2008; De Roos et al. 2005; Engel et al. 2007a; Hardell et al. 1997; Laden et al. 2010; Rothman et al. 1997; Spinelli et al. 2007); fish consumption (Bertrand et al. 2010); employment in farming (De Roos et al. 2005); and lactation status (Laden et al. 2010); among others. Rapid weight loss has been found to increase blood PCB concentrations, and because weight loss may occur as a consequence of the NHL disease state, such an effect could serve as a potential source of bias. Although many of the case–control studies controlled for body mass index (BMI), most were unable to examine the potential effects of weight change over time. However, Spinelli et al. (2007) found that excluding patients with > 10% weight loss before diagnosis had little effect on the observed association between PCB exposure and NHL. Case–control studies that analyzed NHL by subtype (Bertrand et al. 2010; Cocco et al. 2008; Hardell et al. 2009; Morton et al. 2008) found positive associations with PCB exposure for some NHL subtypes; however, the small number of cases makes it difficult to draw meaningful conclusions about potential differences among subtypes. Finally, the studies with positive findings are not restricted to populations in which biological samples were collected from earlier years, when PCB levels in the environment were higher than present concentrations. For example, samples from Spinelli et al. (2007) were collected between 2000 and 2004.

It has been suggested that the development and/or treatment of NHL may affect PCB levels, and thus could explain the observed positive associations between PCB exposure and NHL in the case–control literature (Golden and Kimbrough 2009). However, multiple lines of evidence contradict this hypothesis. First, nested case–control studies, by virtue of study subject selection from within prospective cohorts, demonstrate a temporal relationship between measured blood PCB levels and subsequent NHL diagnosis which obviates the issue of biases from disease or treatment effects (Bertrand et al. 2010; Engel et al. 2007a; Laden et al. 2010; Rothman et al. 1997). Moreover, many of the population-based case–control studies collected samples before chemotherapy or restricted analyses to samples collected pre-treatment, effectively eliminating any effect of treatment on measured PCB levels (Colt et al. 2009; De Roos et al. 2005; Hardell et al. 1996, 1997, 2001, 2009; Spinelli et al. 2007). Finally, case–control studies that quantified PCB exposure from residential carpet dust samples, which are less likely to be biased by individual biological modifiers such as age, lactation, BMI, metabolism, and disease effects, have shown associations between elevated PCB levels and NHL (Colt et al. 2005, 2009; Morton et al. 2008). In these studies, although carpet dust samples were collected postdiagnosis, the sampled carpets were at least 5 years old, and because PCBs are resistant to sun, moisture, and microorganisms, the presence of PCBs in carpet dust is indicative of past chronic exposures (Colt et al. 2005).

*Cohort studies.* A series of studies funded by the National Institute for Occupational Safety and Health (NIOSH) examined mortality rates in a cohort of capacitor manufacturing workers at plants in New York and Massachusetts (Brown 1987; Brown and Jones 1981; Prince et al. 2006a, 2006b). The earliest studies (Brown 1987; Brown and Jones 1981) did not observe elevated mortality for the broad category of “lymphatic and hematopoietic malignancies” [see Supplemental Material, Table S2 (http://dx.doi.org/10.1289/ehp.1104652)]. However, slightly elevated standardized mortality ratios (SMR) were observed among females in the Massachusetts plant (observed/expected = 2/1.71; SMR = 1.17, *p* > 0.05), the group that began employment earliest and was employed the longest (Brown and Jones 1981). Studies reporting additional follow-up of the New York facility (Kimbrough et al. 1999, 2003) did not observe increased mortality from lymphatic and hematopoietic malignancies, which was consistent with the earlier analyses by Brown and colleagues. However, two more recent studies, one restricted to workers highly exposed to PCBs (Prince et al. 2006a) and one of an expanded cohort of workers with ≥ 90 days of potential PCB exposure (Prince et al. 2006b), which specifically examined mortality from NHL and included 58 years of follow-up from both plants, found elevated NHL mortality for the total cohort (observed/expected = 10/7.63; SMR = 1.31, 95% CI: 0.63, 2.41) (Prince et al. 2006a), among women (observed/expected = 8/4.30; SMR = 1.86, 95% CI: 0.80, 3.66) (Prince et al. 2006a), and in the Massachusetts plant (observed/expected = 9/4.54; SMR = 1.98, 95% CI: 0.91, 3.77) (Prince et al. 2006a); (observed/expected = 23/17.97; SMR = 1.28, 95% CI: 0.81, 1.92) (Prince et al. 2006b).

A study of capacitor manufacturing workers in Indiana did not observe increased mortality for the broad category of “lymphatic and hematopoietic malignancies” (Sinks et al. 1992). However, after an additional 19 years of follow-up (Ruder et al. 2006), the authors were able to analyze mortality rates of NHL specifically, and observed elevated NHL mortality in the total cohort (observed/expected = 9/7.32; SMR 1.23, 95% CI: 0.60, 2.30) as well as among workers in the middle (observed/expected = 5/2.59; SMR = 1.93, 95% CI: 0.6, 4.5) and highest (observed/expected = 3/2.31; SMR = 1.3, 95% CI: 0.3, 3.8) tertiles of estimated PCB exposure. In a study of capacitor manufacturing workers in Illinois, Mallin et al. (2004) found elevated mortality from lymphosarcoma and reticulosarcoma among white females (SMR 1.53; 95% CI: 0.32, 4.48). Further, in an analysis restricted to 1960–1999, during which time it was possible to recode malignancies as NHL specifically, the authors observed an NHL mortality 76% higher than expected among white females (SMR = 1.76; 95% CI: 0.94, 3.02). A series of reports on Italian capacitor manufacturing workers found elevated mortality from lymphatic and hematologic neoplasms (SMR range: 1.41–4.4) (Bertazzi et al. 1982, 1987; Tironi et al. 1996). A small study of capacitor manufacturing workers in Sweden (Gustavsson and Hogstedt 1997) found elevated NHL incidence (SIR = 1.49; 95% CI: 0.02, 8.30) and mortality (SMR = 2.54; 95% CI: 0.07, 14.2) from malignant lymphomas in the total cohort, although the analyses were based on only one NHL case and one lymphoma death.

A study of transformer manufacturing workers in Ontario, Canada, found elevated mortality for lymphatic and hematopoietic malignancies (SMR = 2.90; 95% CI: 0.3, 12.4) (Liss 1989). Another study of transformer manufacturing workers in Canada found elevated mortality from NHL in the overall cohort, although this analysis was based on only 2 NHL deaths out of 71 total deaths (Yassi et al. 1994). In a study of electric utility workers, Loomis et al. (1997) found that mortality was not increased for lymphosarcoma and reticulosarcoma, but was slightly elevated for “other lymphatic neoplasms” (SMR = 1.04; 95% CI: 0.89, 1.20). A study of fishermen from the east coast of Sweden with historically high intake of fatty fish containing PCBs did not find elevated NHL incidence or mortality (Svensson et al. 1995).

*Comments on cohort studies.* Important methodological features of the occupational cohort studies may cause their results to be biased toward the null. No direct measurement of PCBs in biological samples was used in analyzing disease risk associated with PCB exposure. Instead, exposure was estimated based on job titles, work history, or job–exposure matrices, possibly resulting in exposure misclassification. Additionally, because studies conducted in the past are typically limited to the older diagnostic criteria and outcome definitions available at the time of the study (or at the time of the deaths observed in the study), many occupational cohort studies examined only broad classifications such as “lymphatic and hematologic malignancies,” and were not able to perform analyses specific to NHL. Indeed, studies with null findings were generally those that examined such larger outcome categories. In contrast, the studies that specifically examined NHL as a diagnosis or cause of death generally observed associations with exposure (Gustavsson and Hogstedt 1997; Mallin et al. 2004; Prince et al. 2006a; Ruder et al. 2006; Yassi et al. 1994). Comparisons of mortality in occupational cohorts to expectations for the general population may be influenced by the healthy worker effect—a phenomenon that occurs because employed populations generally have lower rates of disease than the general population, thereby making any elevation in disease risk caused by occupational exposure more difficult to detect when using the general population as a comparison group (Rothman et al. 2008).

Furthermore, most of the occupational cohort studies were underpowered to detect associations with NHL. Many focused on mortality, missing incident NHL cases that did not result in death. Studies that included incident cancer cases had few cases due to the relative rarity of lymphatic and hematologic malignancies, and specifically of NHL. Many of the workers in these studies were employed only for short durations and/or left employment at relatively young ages, thereby limiting the size of the at-risk population [see Supplemental Material, Table S2 (http://dx.doi.org/10.1289/ehp.1104652)].

An important factor that may affect the external validity of the occupational cohort studies is the difference in the nature of the PCB mixtures in occupational settings versus environmentally bioaccumulated mixtures. Industrial PCB mixtures have been found to be more rapidly metabolized and less toxic than environmentally persistent mixtures of PCBs (U.S. EPA 1996). Furthermore, PCB elimination is affected by exposure dose, with shorter PCB half-lives observed among capacitor manufacturing workers with higher baseline serum PCB levels compared to workers with lower baseline levels (Phillips et al. 1989). Therefore, the assumption that occupational cohort studies should demonstrate increased risks of NHL in the case of a “true” causal relationship fails to consider the differences in exposure route, dose, metabolism, and congener mixture composition.

## PCBs, NHL, and the Immune System

A common theme of immune dysregulation unifies many of NHL’s strongest risk factors, including congenital or acquired immunodeficiency syndromes, such as HIV/AIDS; iatrogenic immune suppression, such as occurs following organ transplantation; and autoimmune diseases such as rheumatoid arthritis, systemic lupus erythematosus, and Sjögren’s syndrome (Grulich et al. 2007). Any factor that triggers immunosuppression can create a permissive environment in which malignancy is more likely to occur by impairing immunosurveillance and other protective mechanisms that lead to the eradication of malignant cells. Additionally, chronic antigenic stimulation results in B-cell proliferation and compensatory down-regulation of T cell–mediated immunity. This state of heightened B-cell proliferation increases the risk of random genetic errors that can render cells malignant, and the down-regulation of T-cell response results in an immunosuppressive state (Fisher and Fisher 2004). Several authors have posited that even subtle immune dysregulation may increase NHL risk by decreasing host resistance, reducing control of cellular proliferation and differentiation, and diminishing tumor surveillance mechanisms (Chow and Holly 2002; Fisher and Fisher 2004; Grulich et al. 2007).

Increasing evidence suggests that PCBs can cause dysregulation of the immune system through immunosuppression and immune stimulation/inflammation, both of which have been implicated in the molecular pathogenesis of NHL (Fisher and Fisher 2004), thus leading to the hypothesis that the observed association between PCBs and NHL may be mediated by immune effects. Although direct evidence that links PCBs, immune dysregulation, and NHL is limited, several indirect lines of evidence support immune dysregulation as a means by which PCBs may cause NHL.

*Immune suppression.* Epidemiological studies show that PCBs are associated with modification of both innate and adaptive immunity, including effects on immune cells and signaling molecules, with implications for both immune response and initiation. Such effects are manifested as an increased incidence of infections; insufficient antibody response to vaccination; and changes in immune organs, lymphocyte subsets, and lymphocyte function.

PCB exposure, as estimated by various measures including PCB levels in cord blood, maternal sera, and breast milk, has been associated with an increased incidence of respiratory infections, ear infections, influenza, and chicken pox in healthy Dutch (Weisglas-Kuperus et al. 2000, 2004) and Inuit (Dallaire et al. 2006; Dewailly et al. 2000) preschoolers; children of capacitor manufacturing workers, particularly those breast-fed for lengthy periods (Hara 1985); and children prenatally exposed to PCB- and PCDF-contaminated rice oil during the Yusho and Yu-Cheng poisoning incidents (Chao et al. 1997; Guo et al. 2004; Yu et al. 1998).

PCB exposure has also been associated with insufficient response to vaccination. In two birth cohorts from the Faroe Islands with pre- and postnatal PCB exposure from dietary consumption of whale blubber, insufficient antibody response to diphtheria and tetanus toxoid was associated with PCB exposure (Heilmann et al. 2006, 2010), with each doubling of cumulative PCB exposure associated with a 24% reduction in diphtheria antibody response at 18 months of age and each doubling of prenatal PCB exposure associated with a 16% reduction in tetanus toxoid response at 7 years of age (Heilmann et al. 2006). By 5 years of age, a doubling of PCB exposure was associated with a 30% increased odds of antidiptheria toxoid levels below the limits for long-term protection (Heilmann et al. 2010).

Studies in both adults and children have demonstrated an association between PCB exposure and reduced functional capacity of lymphocytes, as indicated by decreased responses to mitogen stimulation (Belles-Isles et al. 2002; Bilrha et al. 2003; Daniel et al. 2001; Nakanishi et al. 1985) and decreased delayed-type hypersensitivity reactions (Lu and Wu 1985; Van Den Heuvel et al. 2002). Additionally, studies have demonstrated correlations between PCB exposure or markers of PCB exposure and alterations to normal lymphocyte subpopulations. Hagmar et al. (1995) observed dose-related inverse associations between fatty fish consumption, an important source of exposure to PCBs and other persistent organic pollutants (POPs) in certain areas of the world, and the proportion of cytotoxic T cells in Latvian villagers. Daniel et al. (2001) found that a greater proportion of individuals with elevated blood levels of PCB-101 had low DR+ cell counts (a measure of antigen-presenting cells) among 146 workers occupationally exposed to PCBs for a minimum of 6 months. Lawton et al. (1985) studied 194 capacitor workers exposed to Aroclors (industrial mixtures of PCBs) before and after the plant discontinued use of PCBs and found abnormally high lymphocyte counts, defined as > 2 SDs from the internal age- and sex-adjusted standards used by the clinical laboratory, among workers before PCB discontinuation. Correlations between PCB exposure and lymphocyte subsets have been observed among Dutch schoolchildren exposed to background levels of PCBs measured in maternal, cord, and infant blood (Weisglas-Kuperus et al. 1995, 2000). Studies of other groups exposed to multiple POPs, including PCBs, have also observed differences in lymphocyte subsets relative to comparison groups, including breast-fed Japanese (Nagayama et al. 1998) and Inuit babies (Dewailly et al. 2000) and newborns in a Quebec subsistence fishing community (Belles-Isles et al. 2002).

Animal studies conducted in rodents and nonhuman primates exposed to PCBs and dioxins have observed similar outcomes to studies of humans, including suppressed antibody response to immunization with sheep red blood cells and reduced response to mitogen in lymphocyte proliferation assays, and provide a convincing body of evidence for the immunosuppressive effects of Aroclors and DL-PCBs [as reviewed by Kerkvliet (2009), Selgrade (2007), and Tryphonas (1995)]. Bone marrow hypocellularity and atrophy of lymphoid organs, including the thymus and spleen, have been observed in many animal species following oral, dermal, and transplacental exposure to high- and low-dose PCB mixtures and certain congeners [Kimbrough et al. 1978; National Toxicology Program (NTP) 2006b; Smialowicz et al. 1989]. Consistent with these findings, the only study in humans to examine PCB exposure and thymus growth found that among a cohort of 982 mother–infant pairs from a PCB-contaminated region in Eastern Slovakia, prenatal PCB exposure, assessed through maternal serum PCB levels, was associated with significantly reduced thymus volume at birth (*p* = 0.047) (Park et al. 2008).

*Immunosurveillance and antigen recognition.* Human studies have provided evidence supporting the effects of PCBs on several markers of normal immunosurveillance and tumor control. Among these markers, NK cells are particularly important in cancer immunosurveillance because of their ability to eliminate early malignant cells without activation by CD4 cells. A study of heavy fatty-fish consumers and nonconsumers in Sweden (Svensson et al. 1994) found reduced levels of NK cells in the peripheral blood of fatty-fish consumers and a significant negative correlation between weekly fish intake and proportions of NK cells. In a subsample of 11 participants, significant negative correlations were observed between number of NK cells and blood levels of PCBs 126 (*p* = 0.02) and 118 (*p* = 0.01). Although no associations were observed between levels of organic mercury in erythrocytes and any of the lymphocyte subsets, the effect of concomitant exposure to mercury and n-3 fatty acids in this population cannot be discounted. A similar study conducted in Latvia among low, intermediate, and high fish consumers (Hagmar et al. 1995) found dose-related inverse associations between fish consumption and proportion of NK cells.

Victims of the Yu-Cheng PCB/PCDF poisoning were found to have fewer monocytes and polymorphonuclear leukocytes (PMNs) bearing immunoglobulin and complement receptors (Chang et al. 1982), and among 146 workers occupationally exposed to PCBs for a minimum of 6 months, blood levels of PCB-138 above the mean in healthy controls were associated with a greater frequency of undetectable levels of interleukin (IL)-4 (Daniel et al. 2001). Reductions in these early innate immune response cells, including monocytes, PMNs, and NK cells, can impair the ability of the immune system to identify foreign antigens and limit the early cytokine and chemokine signaling that initiates a robust and complete immune response (Murphy et al. 2008). T cells also play a role in cancer immunosurveillance, and PCBs have been shown to affect T-cell number, function, and maturation (Belles-Isles et al. 2002; Bilrha et al. 2003; Nagayama et al. 1998; Weisglas-Kuperus et al. 2000).

Mammalian and *in vitro* studies examining the effect of dioxins on macrophages, neutrophils, and dendritic cells indicate that DL-PCBs can affect the innate immune system (Kerkvliet 2009). Though toxicological studies on NDL-PCBs are in their infancy, recent *in vitro* data demonstrate the ability of NDL-PCBs to suppress the macrophage response and support an apoptotic mechanism initiated by PCBs 101, 153, and 180 (Ferrante et al. 2011). Additionally, Levin et al. (2005) showed that exposure of healthy human leukocytes to the NDL-PCBs 138, 153, and 180 resulted in reduced phagocytosis mediated through both reduced neutrophil and monocyte activity, the effects of which were more pronounced with exposure to mixtures of multiple NDL-PCBs. In contrast, exposure to the DL-PCB-169 or to 2,3,7,8-tetrachlorodibenzo-*p*-dioxin (TCDD) did not affect phagocytosis, suggesting an independent immunotoxic effect of NDL-PCBs.

*Immune stimulation.* Immune stimulation can result in hypersensitivity reactions to antigens that create an unregulated immune response, including a failure of the immune system to recognize “self,” which may be indicated by increased risk of allergic, autoimmune, and/or inflammatory disease. Chronic abnormal lymphocyte activation is thought to predispose to NHL, and risks of NHL among patients with autoimmune disease are highly elevated (Grulich et al. 2007).

Epidemiological studies have observed associations between exposure to PCBs and increased risks of autoimmune diseases, including systemic lupus erythematosus (Tsai et al. 2007) and rheumatoid arthritis (Lee et al. 2007), that are in turn associated with an increased risk of NHL. Studies of children have found conflicting associations between PCB exposure and both increased (Hara 1985; Van Den Heuvel et al. 2002; Yu et al. 1998) and decreased rates of allergy/atopy (Van Den Heuvel et al. 2002; Weisglas-Kuperus et al. 2000, 2004; Yu et al. 1998).

Although the epidemiological literature is limited regarding the effect of PCBs on indicators of immune stimulation, the toxicological literature demonstrates strong evidence for an effect of PCBs on inflammation (as reviewed by Strauss and Heiger-Bernays 2012). PCB-associated inflammation, microscopically identified as aggregates of immune cells, has been observed in many animal studies, including chronic rodent feeding studies conducted on the carcinogenicity of PCBs (Kimbrough et al. 1972; NTP 2006b). Mechanistic studies indicate that DL-PCBs target the acute phase of inflammation, where macrophages recognize patterns on foreign biological substances through the activation of toll-like receptors that initiate various signaling pathways leading to production of proinflammatory cytokines, such as IL-6, tumor necrosis factor (TNF)–α and macrophage inflammatory protein (MIP)–2 (Hennig et al. 2002; Kerkvliet 2009).

*Dioxin-like and non-dioxin-like activity.* The coplanar and mono-*ortho*-substituted PCBs (DL-PCBs) act through a mechanism of toxicity similar to that of dioxins, which exert potent immunosuppressive effects by binding to the aryl hydrocarbon receptor (AhR) (Kerkvliet 2009). TCDD, the dioxin that is the most potent AhR ligand, is considered to be a known human carcinogen and is linked specifically with increased risk of NHL [International Agency for Research on Cancer (IARC) 1997; NTP 2011]. In a large, population-based case–control study of 422 NHL cases and 459 controls, Ng et al. (2010) identified a significant interaction (*p* = 0.013) between a single nucleotide polymorphism (SNP) in the AhR gene and increasing quartiles of PCB-118, thus suggesting that AhR gene variants may modify the effect of PCBs on risk of NHL.

Although the AhR mechanism is the best studied, recent research also demonstrates immunotoxic effects of NDL-PCBs, which occur independently of AhR (Duffy and Zelikoff 2006; Ferrante et al. 2011; Levin et al. 2005; Lyche et al. 2004). Several indirect mechanisms have been proposed, such as signaling via the neural-immune axis, perhaps by affecting calcium homeostasis or serotonergic systems (Duffy-Whritenour et al. 2010; Pessah et al. 2010).

*Interaction with Epstein-Barr virus (EBV).* EBV is a B cell–transforming virus that establishes a latent infection, which is controlled by T lymphocyte–mediated mechanisms in the host (Thorley-Lawson and Gross 2004), and is causally linked with at least one NHL subtype, Burkitt’s lymphoma (Mueller et al. 1992). Because approximately 90% of the worldwide population is infected with EBV by adulthood, exposure to EBV alone is unlikely to be a sufficient cause of NHL in the general population. However, several case–control studies provide evidence for biological interaction between blood levels of PCBs and titers of antibodies to Epstein-Barr virus early antigen (EBV-EA) and risk of NHL (Hardell et al. 2001, 2009; Nordstrom et al. 2000; Rothman et al. 1997) [see Supplemental Material, Table S3 (http://dx.doi.org/10.1289/ehp.1104652)]. In these studies, when PCB levels and EBV-EA titers were dichotomized at the median level among controls, the ORs for NHL in the high-PCB/high-EBV category ranged from 3.0 to 22.3. All except the lowest of these ORs [OR = 3.0; 95% CI: 1.2, 7.8; observed for lower chlorinated PCBs in the study by Hardell et al. (2009)] are greater than would be expected under biologic independence (Rothman et al. 2008); however, most are based on small numbers of observations. The ability of PCBs to suppress T lymphocyte–mediated immunity provides a plausible explanation for the evidence suggesting interactive effects of EBV-EA titers and PCB levels on NHL risk seen in the epidemiological literature (Vineis et al. 1992). An immunosuppressive state may allow transforming viruses to proliferate, potentially causing malignant transformation through integration of viral DNA into host genomes and/or triggering antigenic stimulation, thereby increasing the likelihood of genetic mutations in B cells (Fisher and Fisher 2004).

## Conclusions and Future Directions

The key issues that have influenced conclusions of earlier authors and expert panels regarding PCB–NHL causality are largely derived from “conflicting” findings from the occupational cohort studies and a lack of an animal model for PCB-induced lymphomagenesis. Although PCB exposures in the occupational cohort studies are generally higher than those experienced by the general population, these studies are limited by methodological shortcomings for evaluating NHL risks and a potential lack of applicability to exposures sustained by the general population due to differences in exposure route, dose, metabolism, and congener mixture composition. Regarding animal models for PCB-induced lymphoma, Strauss and Heiger-Bernays (2012) reviewed the inadequacy of existing PCB bioassays for detecting NHL. Reevaluation of early PCB rat bioassays revealed previously overlooked evidence of NHL (Strauss and Heiger-Bernays 2012), and recent bioassays with updated histopathological protocols have observed cases of lymphoma and leukemia in PCB-exposed rats and controls (NTP 2006a, 2006b). Studies using a mouse model and with updated histopathological protocols, yet to be conducted, would be better suited to evaluate the relationship between PCBs and NHL in animal models (Strauss and Heiger-Bernays 2012).

Overall, we conclude that the weight of evidence supports a causal role of PCBs in NHL carcinogenesis, with the strongest evidence for this association coming from case–control studies conducted among the general population, including several prospective nested case–control studies. Furthermore, epidemiological and toxicological data show that PCBs have immunosuppressive and immunostimulatory effects. Because severe immune dysregulation is a strong risk factor for NHL, it is plausible that more subtle immune dysregulation is also a means by which PCBs can cause NHL.

Several areas of research may further elucidate the relationship between PCBs and NHL. Numerous studies have been published in the last decade identifying genetic risks for NHL (e.g., Hosgood et al. 2011; Morton et al. 2008; Shen et al. 2011; Skibola et al. 2007), and future areas of study should include the influence of PCBs and other POPs on genetic and epigenetic mechanisms underlying cancer development. For example, studies of the SEER NHL case–control population have demonstrated evidence of an interaction between organochlorine levels in blood and carpet dust and several potential susceptibility loci in immune and inflammatory genes (Colt et al. 2009; Wang et al. 2007), indicating that common immune gene variants with low penetrance may enhance susceptibility to NHL and modify the effect of exposure to organochlorines on risk of NHL in the general population. Also, a recent study of 84 healthy Koreans found significant positive correlations between background levels of serum PCBs and increasing telomere length in peripheral leukocytes, which the authors suggest may indicate that general population levels of PCBs may act as a tumor promoter for hematopoietic malignancies (Shin et al. 2010). Studies of the immunotoxicity of NDL-PCBs lag behind those of DL-PCBs, but it is clear that NDL-PCBs can have potent effects on the immune system (Duffy and Zelikoff 2006; Levin et al. 2005; Lyche et al. 2004), which is particularly relevant because NDL-PCBs comprise the bulk of environmental and bioaccumulated PCBs. Recent reviews (IARC 2010b; Robertson and Ludewig 2011) have highlighted the need for greater research into airborne and other continuing sources of exposure to PCBs. Airborne PCBs are more readily metabolized and therefore are not generally found in biological samples, but can result in bioactivated intermediates that act as genotoxins, mutagens, and carcinogens.

PCBs as a class are currently classified as probably or reasonably anticipated to be human carcinogens (IARC 2010a; NTP 2011; U.S. EPA 1996), although DL-PCB-126 was recently classified as a known human carcinogen (IARC 2010a, 2010c). PCB carcinogenicity was last fully reviewed by the NTP in 1981, the IARC in 1987, and the U.S. EPA in 1996. We believe that consideration of the methodological issues reviewed here—with an emphasis on the more methodologically robust case–control studies published since the last formal expert panel reviews of PCB carcinogenicity, as well as increasing evidence for immune dysregulation as a means by which PCBs may cause NHL—establishes the basis for an updated assessment of the carcinogenicity of PCBs by relevant national and international scientific panels.

## Supplemental Material

(672 KB) PDF
