# Supplementary material for: Current Status of the Epidemiologic Evidence Linking Polychlorinated Biphenyls and Non-Hodgkin Lymphoma, and the Role of Immune Dysregulation
Source: Environ Health Perspect. 2012 May 2;120(8):1067–75. doi: 10.1289/ehp.1104652 (PMC3440083; doi:10.1289/ehp.1104652)
Supplement: (672 KB) PDF [file ehp.1104652.s001.pdf]

Supplemental Material

Current Status of the Epidemiologic Evidence Linking Polychlorinated Biphenyls and Non-Hodgkin Lymphoma, and the Role of Immune Dysregulation

Shira Kramer,[1] Stephanie Moller Hikel,[1] Kristen Adams,[1] David Hinds,[1] Katherine Moon [1]

[1] Epidemiology International, Hunt Valley, Maryland, USA

Supplemental Material, Table of Contents.

Literature Search Key Words	p. 3
Summary of Inclusion/Exclusion Criteria for PCB-NHL Weight of Evidence Analysis	p. 4
Figure S1. Results of Literature Search for PCB-NHL Weight of Evidence Analysis.	p. 5
Table S1. Results from Case-Control Studies of the Association Between PCB Exposure and NHL	p. 6
Table S2. Characteristics of Cohort Studies Examining the Association Between Occupational PCB Exposure and NHL or Lymphoma	p. 17
Table S3. Interaction Between PCBs and EBV and Risk of NHL	p. 20
References	p. 21

Supplemental Material, Literature Search Key Words.

Question 1: PCB-NHL Weight of Evidence Analysis

Key words and indexing terms included relevant combinations of the following terms: “polychlorinated biphenyl”, “PCB”, “Aroclor”, “Arochlor”, “Clophen”, “organochlorine”, “aryl hydrocarbon”, “AhR”, “dioxin-like”, “lymphoma”, “non-Hodgkin”, “NHL”, “lymphohematopoietic”, “hematologic”, “lymphatic”, “lymphosarcoma”, “reticulosarcoma”, “carcinogen”, “cancer”, and “carcinogenicity”.

Question 2: Evidence for the Role of Immune Dysregulation in Linking PCB Exposure and NHL Risk.

Key words and indexing terms included relevant combinations of the following terms: “polychlorinated biphenyl”, “PCB”, “Aroclor”, “Arochlor”, “Clophen”, “organochlorine”, “dioxin-like”, “immune”, “inflammation”, “autoimmune”, “immunotoxic”, “immunodeficiency”, “Epstein Barr virus”, “EBV”, “antibody”, and “infection”.

Supplemental Material, Summary of Inclusion/Exclusion Criteria for PCB-NHL Weight of Evidence Analysis.

The titles, abstracts, and full text (when necessary) of all citations resulting from our literature search were reviewed for potential inclusion in the weight of evidence analysis, based on the following inclusion criteria:

1) English language or English translation available
2) Humans as the primary subject (versus animal, toxicological, mechanistic, and/or methodology as the primary subject)
3) Presentation of original data (versus reviews, commentaries, or reports)
4) Specific to PCB exposure
5) Specific to NHL or lymphoma as an outcome
6) Statistical analysis of the main effect of PCB exposure on NHL/lymphoma, including data on magnitude of the effect (versus studies performing no statistical analyses or presenting only p-values)
7) For abstracts only: Not subsequently published in manuscript form

Figure S1 presents the numbers of citations excluded based upon each of the above-listed criteria. The relevance of each citation to the inclusion criteria was evaluated in the order of the criteria listed above, and the number of citations excluded for each criterion was counted. It was possible for a particular citation to fail to meet multiple of the inclusion criteria; however, it was only counted under the first criterion which it failed to meet.

Supplemental Material, Figure S1. Results of Literature Search for PCB-NHL Weight of Evidence Analysis.

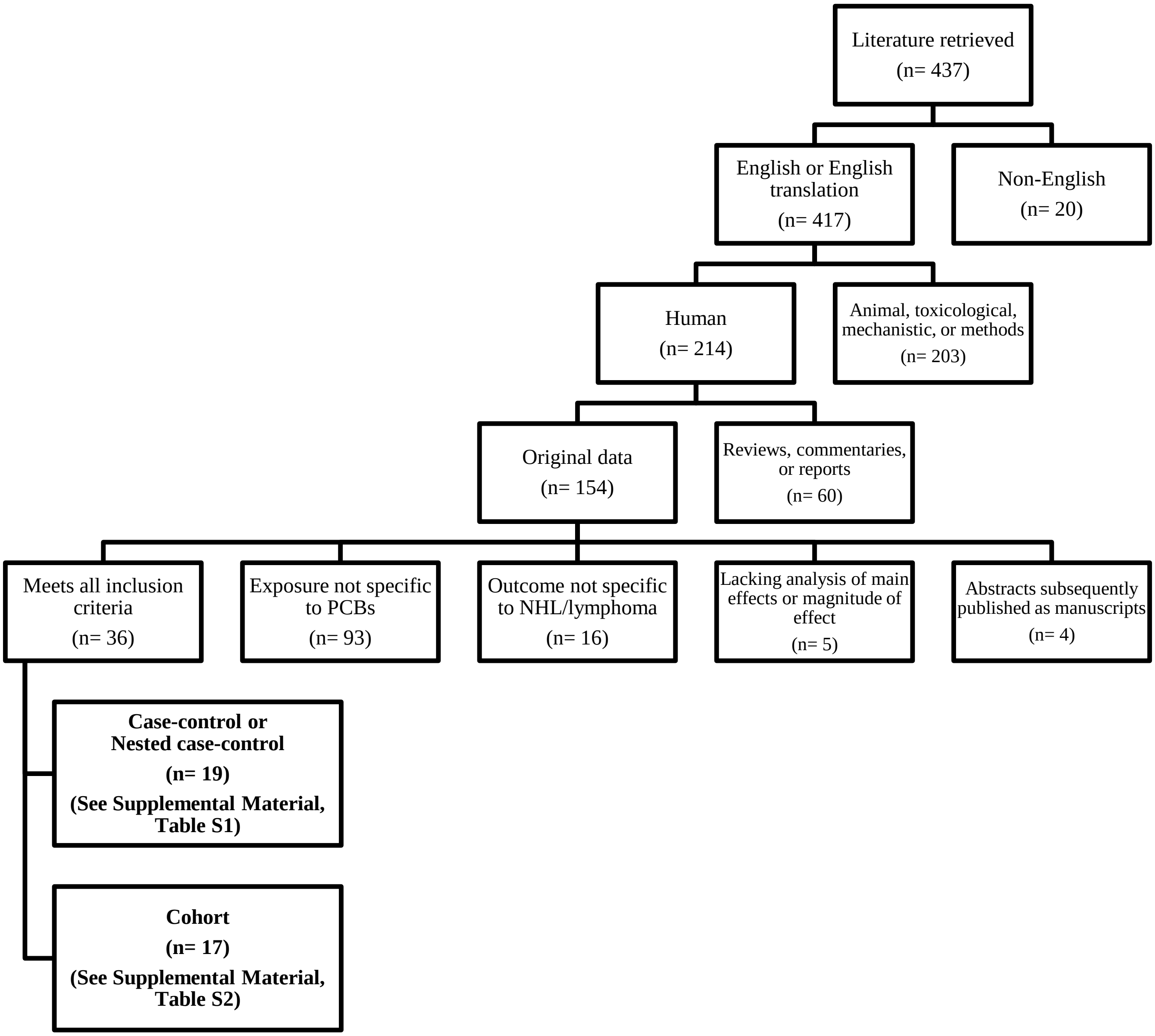

Supplemental Material, Table S1. Results from Case-Control Studies of the Association Between PCB Exposure and NHL

**Citation** Study Population	**PCB source**	**Control Matching Variables**	**PCB Congener or Group**	**Exposure Level (lipid adjusted)**	**# Cases**	**# Controls**	**OR** [a]	**95% CI**		**p-value**	**Adjustment Variables** [b]
**Bertrand et al, 2010** Physicians' Health Study	Plasma	Race, age, timing of blood sample, fasting status at blood draw	Σ PCBs	163-167 ng/g lipid	33	81	1.0	reference		**trend <0.01**	Region, BMI, smoking status, alcohol intake, height
				>167-742 ng/g lipid	31	82	0.86	0.47	1.6		
				>742-894 ng/g lipid	34	82	0.99	0.55	1.8		
				>894-1121 ng/g lipid	46	82	1.3	0.71	2.3		
				>1121-5322 ng/g lipid	61	82	1.6	0.91	2.9		
			Σ Immunotoxic PCBs	24-113 ng/g lipid	32	81	1.0	reference		trend 0.09	
				>113-145 ng/g lipid	35	82	0.98	0.54	1.8		
				>145-189 ng/g lipid	36	82	0.99	0.55	1.8		
				>189-245 ng/g lipid	45	82	1.2	0.64	2.1		
				>245-1813 ng/g lipid	57	82	1.4	0.80	2.6		
			Σ PCB 118, 138, 153, 180	35-275 ng/g lipid	31	81	1.0	reference		**trend <0.01**	
				>275-344 ng/g lipid	32	82	0.96	0.53	1.8		
				>344-423 ng/g lipid	36	82	1.1	0.60	2.0		
				>423-525 ng/g lipid	38	82	1.1	0.59	2.1		
				>525-2440 ng/g lipid	68	82	1.8	1.0	3.2		
			PCB 118	4.0-42 ng/g lipid	33	81	1.0	reference		trend 0.15	
				>42-56 ng/g lipid	29	82	0.8	0.42	1.5		
				>56-77 ng/g lipid	40	82	1.1	0.59	2.0		
				>77-105 ng/g lipid	46	82	1.2	0.63	2.2		
				>105-734 ng/g lipid	57	82	1.4	0.76	2.5		
			PCB 138	4.0-59 ng/g lipid	29	81	1.0	reference		**trend 0.02**	
				>59-76 ng/g lipid	38	82	1.3	0.68	2.3		
				>76-97 ng/g lipid	38	82	1.2	0.64	2.1		
				>97-122 ng/g lipid	37	82	1.2	0.64	2.2		
				>122-541 ng/g lipid	63	82	1.8	0.98	3.2		
			PCB 153	6.1-95 ng/g lipid	28	81	1.0	reference		**trend <0.01**	
				>95-122 ng/g lipid	37	82	1.2	0.67	2.3		
				>122-148 ng/g lipid	36	82	1.3	0.68	2.4		
				>148-188 ng/g lipid	37	82	1.2	0.62	2.2		
				>188-761 ng/g lipid	67	82	**2.1**	**1.1**	**3.8**		
			PCB 180	21-68 ng/g lipid	25	81	1.0	reference		**trend <0.01**	
				>68-84 ng/g lipid	40	82	1.5	0.82	2.7		
				>84-102 ng/g lipid	35	82	1.4	0.75	2.7		
				>102-126 ng/g lipid	44	82	1.8	0.96	3.3		
				>126-528 ng/g lipid	61	82	**2.4**	**1.3**	**4.5**		

Citation Study Population	PCB source	Control Matching Variables	PCB Congener or Group	Exposure Level (lipid adjusted)	# Cases	# Controls	OR [a]	95% CI		p-value	Adjustment Variables [b]
**Cocco et al, 2008** Participants in Epilymph case-control study from France, Germany, and Spain	Plasma	None. Plasma samples for controls randomly sampled from Epilymph study participants	Σ PCBs	≤200.42 ppb	41	51	1.0	reference		trend 0.83	Age, gender, education, study center
				200.43-387.79 ppb	50	51	1.2	0.6	2.2		
				387.80-576.36 ppb	33	50	0.7	0.3	1.4		
				≥576.37 ppb	50	51	1.0	0.5	2.0		
			Σ Immunotoxic PCBs	≤153.46 ppb	38	51	1.0	reference		trend 0.76	
				153.47–215.38 ppb	37	50	1.0	0.5	1.8		
				215.39–334.14 ppb	48	51	1.1	0.6	2.1		
				≥334.15 ppb	51	51	1.1	0.6	2.1		
			Σ BRCA1 inhibitors	≤135.46 ppb	41	51	1.0	reference		trend 0.77	
				135.47-215.38 ppb	31	50	0.8	0.4	1.5		
				215.39-334.14 ppb	55	51	1.3	0.7	2.5		
				≥334.15 ppb	47	51	1.0	0.5	1.9		
			Σ Pseudo-estrogen PCBs	≤83.81 ppb	39	51	1.0	reference		trend 0.63	
				83.82-166.75 ppb	44	50	1.1	0.6	2.1		
				166.76-290.85 ppb	40	51	0.9	0.4	1.8		
				≥290.86 ppb	51	51	1.2	0.6	2.5		
			Σ High chlorinated anti-estrogenic PCBs	≤0.30 ppb	20	22	1.0	reference		trend 0.96	
				0.31-65.63 ppb	42	60	0.9	0.4	1.9		
				65.64-113.43 ppb	49	59	0.9	0.4	2.2		
				≥113.44 ppb	63	62	1.0	0.4	2.2		
			Σ Phenobarbitol-inducer PCBs	≤99.540 ppb	39	60	1.0	reference		trend 0.59	
				99.55-161.85 ppb	46	47	1.5	0.8	2.7		
				161.86-237.29 ppb	35	48	1.0	0.5	2.0		
				≥237.30 ppb	54	48	1.3	0.7	2.5		
			Σ Phenobarbitol and 3-methyl cholanthrene mixed inducer PCBs	≤57.95 ppb	40	54	1.0	reference		trend 0.74	
				57.96-106.03 ppb	41	48	1.4	0.7	2.7		
				106.04-192.91 ppb	56	50	1.5	0.8	2.8		
				≥192.92 ppb	37	51	0.9	0.5	1.8		
			PCB 28	≤10.50 ppb	83	99	1.0	reference		trend 0.23	
				10.51-31.70 ppb	25	34	0.9	0.4	1.8		
				31.71-67.94 ppb	21	34	0.7	0.3	1.5		
				≥67.95 ppb	45	36	1.6	0.8	3.2		
			PCB 118	≤12.30 ppb	94	100	1.0	reference		**trend 0.004**	
				12.31–38.76 ppb	41	34	1.0	0.5	2.0		
				38.77–59.17 ppb	20	34	0.5	0.2	1.0		
				≥59.18 ppb	19	35	0.4	0.2	0.8		
			PCB 138	≤45.73 ppb	51	66	1.0	reference		trend 0.88	
				45.74–72.41 ppb	37	46	1.1	0.6	1.9		
				72.42–116.12 ppb	42	45	1.1	0.6	2.0		
				≥116.13 ppb	44	46	1.1	0.6	2.0		
			PCB 153	≤62.56 ppb	43	65	1.0	reference		trend 0.70	
				62.57-100.66 ppb	51	46	1.5	0.8	2.8		
				100.67-142.43 ppb	28	46	0.8	0.4	1.6		
				≥142.44 ppb	52	46	1.3	0.7	2.5		
			PCB 170	≤0.20 ppb	53	65	1.0	reference		trend 0.83	
				0.21–21.53 ppb	40	46	1.1	0.5	2.2		
				21.54–34.28 ppb	36	45	0.8	0.4	1.7		
				≥34.29 ppb	45	47	1.0	0.5	1.8		
			PCB 180	≤0.30 ppb	23	32	1.0	reference		trend 0.31	
				0.31-51.22 ppb	40	57	1.2	0.6	2.6		
				51.23-85.93 ppb	50	56	1.4	0.6	3.0		
				≥85.94 ppb	61	58	1.5	0.7	3.2		

Supplemental Material, Table S1 (continued).

**Citation** Study Population	**PCB source**	**Control Matching Variables**	**PCB Congener or Group**	**Exposure Level (lipid adjusted)**	**# Cases**	**# Controls**	**OR** [a]	**95% CI**		**p-value**	**Adjustment Variables** [b]
**Colt et al, 2005** NCI-SEER study	Carpet dust	Frequency matched on age, sex, race, study center	Σ PCBs	all below UDL	315	262	1.0	reference		trend 0.14	Study center, sex, age, education
				any above UDL	288	181	**1.5**	**1.2**	**2.0**		
				21.9-82.5 ng/g	88	57	1.4	0.9	2.1		
				82.5-202.8 ng/g	114	63	**1.6**	**1.1**	**2.4**		
				203.8-23,380 ng/g	92	61	1.5	1.0	2.2		
			PCB 105	below UDL	486	368	1.0	reference		trend 0.74	
				above UDL	117	75	1.2	0.9	1.7		
				20.8-31.0 ng/g	40	24	1.3	0.8	2.2		
				31.3-73.0 ng/g	43	26	1.4	0.8	2.4		
				75.8-3,860 ng/g	34	25	1.1	0.6	1.9		
			PCB 138	below UDL	398	305	1.0	reference		trend 0.95	
				above UDL	205	138	1.3	1.0	1.7		
				20.8-33.6 ng/g	64	46	1.2	0.8	1.8		
				33.7-84.2 ng/g	83	46	**1.6**	**1.1**	**2.4**		
				84.9-10,200 ng/g	58	46	1.1	0.7	1.7		
			PCB 153	below UDL	351	280	1.0	reference		trend 0.34	
				above UDL	252	163	**1.4**	**1.1**	**1.8**		
				20.8-32.9 ng/g	74	54	1.2	0.8	1.7		
				33.1-74.2 ng/g	101	55	**1.7**	**1.2**	**2.5**		
				74.5-6,460 ng/g	77	54	1.3	0.9	2.0		
			PCB 170	below UDL	509	389	1.0	reference		trend 0.17	
				above UDL	94	54	1.5	1.0	2.1		
				20.8-28.3 ng/g	30	18	1.4	0.8	2.6		
				28.6-52.9 ng/g	31	18	1.5	0.8	2.7		
				53.7-1,380 ng/g	33	18	1.5	0.8	2.7		
			PCB 180	below UDL	432	338	1.0	reference		**trend 0.03**	
				above UDL	171	105	**1.5**	**1.1**	**2.0**		
				20.8-31.6 ng/g	52	35	1.3	0.8	2.1		
				31.8-54.5 ng/g	55	35	1.5	0.9	2.3		
				55.3-2,870 ng/g	64	35	**1.7**	**1.1**	**2.6**		
**Colt et al, 2009** NCI-SEER study	Carpet dust	Frequency matched on age, sex, race, study center	PCB 180	per 10% increase in exposure (ng/g)	682	513	RR= 0.7%	0.0%	1.3%	**p=0.041**	Age, sex, race, education, study center
	Plasma		PCB 180	per 10% increase in exposure (pg/g lipid)	100	100	RR= 8.3%	1.9%	14.6%	**p=0.009**	
			Σ TEQ	per 10% increase in exposure (pg/g lipid)	96	95	RR= 7.8%	1.1%	17.2%	**p=0.022**	

Supplemental Material, Table S1 (continued).

Citation<br>Study Population	PCB source	Control Matching Variables	PCB Congener or Group	Exposure Level (lipid adjusted)	# Cases	# Controls	OR [a]	95% CI		p-value	Adjustment Variables [b]
**De Roos et al, 2005**<br>NCI-SEER study	Plasma	Age, date of blood draw, sex, study site	Σ Noncoplanar PCBs	≤0.445 mol/g lipid	19	22	1.0	reference		trend 0.24	Age, sex, study site, date of blood draw
				>0.445-0.667 mol/g lipid	21	25	1.30	0.51	3.35		
				>0.667-0.900 mol/g lipid	25	24	1.66	0.61	4.53		
				>0.900 mol/g lipid	27	23	1.85	0.67	5.14		
				per 0.01 mol/g lipid	--	--	*1.003*	*0.996*	*1.010*		
			Σ Low chlorinated PCBs	≤0.027 mol/g lipid	25	26	1.0	reference		trend 0.66	
				>0.027-0.046 mol/g lipid	28	25	1.12	0.51	2.45		
				>0.046-0.066 mol/g lipid	16	24	0.73	0.30	1.75		
				>0.066 mol/g lipid	31	25	1.26	0.52	3.03		
				per 0.01 mol/g lipid	--	--	*1.05*	*0.95*	*1.15*		
			Σ Moderately chlorinated PCBs	≤0.385 mol/g lipid	19	23	1.0	reference		trend 0.29	
				>0.385-0.599 mol/g lipid	25	23	1.52	0.58	4.01		
				>0.599-0.785 mol/g lipid	20	24	1.43	0.49	4.11		
				>0.785 mol/g lipid	29	24	1.88	0.67	5.26		
				per 0.01 mol/g lipid	--	--	*1.003*	*0.996*	*1.011*		
			Σ High chlorinated PCBs	≤0.018 mol/g lipid	17	25	1.0	reference		**trend 0.04**	
				>0.018-0.026 mol/g lipid	24	25	1.59	0.62	4.04		
				>0.026-0.036 mol/g lipid	20	25	1.35	0.53	3.48		
				>0.036 mol/g lipid	37	25	**2.68**	**1.04**	**6.90**		
				per 0.01 mol/g lipid	--	--	*1.17*	*0.99*	*1.39*		
			Σ PCB TEQ	≤6.40 pg/g lipid	28	24	1.0	reference		trend 0.15	
				>6.40-8.69 pg/g lipid	16	25	0.59	0.25	1.40		
				>8.69-13.17 pg/g lipid	20	23	0.86	0.38	1.98		
				>13.17 pg/g lipid	33	24	1.51	0.62	3.67		
				per 10 pg/g lipid	--	--	*1.34*	*0.85*	*2.12*		
			PCB 74	≤7.8 ng/g lipid	25	26	1.0	reference		trend 0.66	
				>7.8-13.3 ng/g lipid	28	25	1.12	0.51	2.45		
				>13.3-19.3 ng/g lipid	16	24	0.73	0.30	1.75		
				>19.3 ng/g lipid	31	25	1.26	0.52	3.03		
				per 10 ng/g lipid	--	--	*1.16*	*0.85*	*1.59*		
			PCB 99	≤5.6 ng/g lipid	24	25	1.0	reference		trend 1.00	
				>5.6-9.3 ng/g lipid	22	25	0.63	0.24	1.68		
				>9.3-16.1 ng/g lipid	30	25	1.04	0.45	2.39		
				>16.1 ng/g lipid	24	25	0.77	0.28	2.10		
				per 10 ng/g lipid	--	--	*0.94*	*0.76*	*1.17*		
			PCB 118	≤8.1 ng/g lipid	29	24	1.0	reference		trend 0.88	
				>8.1-11.8 ng/g lipid	14	25	0.36	0.13	0.98		
				>11.8-25.8 ng/g lipid	30	24	0.91	0.42	1.98		
				>25.8 ng/g lipid	24	24	0.73	0.29	1.84		
				per 10 ng/g lipid	--	--	*0.98*	*0.82*	*1.18*		
			PCB 126	≤18.9 pg/g lipid	29	25	1.0	reference		trend 0.54	
				>18.9-30.3 pg/g lipid	20	25	0.65	0.29	1.49		
				>30.3-52.7 pg/g lipid	21	25	0.73	0.31	1.72		
				>52.7 pg/g lipid	30	24	1.09	0.49	2.41		
				per 10 pg/g lipid	--	--	*1.02*	*0.95*	*1.10*		
			PCB 156	≤5.5 ng/g lipid	17	22	1.0	reference		**trend 0.03**	
				>5.5-7.8 ng/g lipid	27	30	1.70	0.48	6.03		
				>7.8-9.8 ng/g lipid	16	26	1.02	0.32	3.26		
				>9.8 ng/g lipid	40	22	2.70	0.97	7.50		
				per 10 ng/g lipid	--	--	*1.69*	*0.91*	*3.11*		

Supplemental Material, Table S1 (continued).

**Citation** Study Population	**PCB source**	**Control Matching Variables**	**PCB Congener or Group**	**Exposure Level (lipid adjusted)**	**# Cases**	**# Controls**	**OR [a]**	**95% CI**		**p-value**	**Adjustment Variables [b]**
**De Roos et al, 2005 (continued)**				≤18.5 pg/g lipid	22	26	1.0	reference			
				>18.5-28.4 pg/g lipid	23	24	1.14	0.49	2.66	trend 0.11	
			PCB 169	>28.4-37.7 pg/g lipid	20	25	1.08	0.41	2.82		
				>37.7 pg/g lipid	35	24	2.62	0.88	7.80		
				per 10 pg/g lipid	--	--	***1.47***	***1.13***	***1.91***		
				≤25.2 ng/g lipid	26	25	1.0	reference			
				>25.2-38.3 ng/g lipid	20	25	0.82	0.38	1.78	trend 0.53	
			PCB 138-158	>38.3-55.5 ng/g lipid	25	25	1.04	0.47	2.33		
				>55.5 ng/g lipid	29	25	1.22	0.49	3.05		
				per 10 ng/g lipid	--	--	*1.02*	*0.93*	*1.12*		
				≤4.4 ng/g lipid	20	28	1.0	reference			
				>4.4-6.0 ng/g lipid	24	23	1.06	0.36	3.08	trend 0.17	
			PCB 146	>6.0-8.7 ng/g lipid	24	24	1.37	0.50	3.79		
				>8.7 ng/g lipid	32	25	1.81	0.70	4.64		
				per 10 ng/g lipid	--	--	*1.54*	*0.81*	*2.95*		
				≤37 ng/g lipid	23	25	1.0	reference			
				>37-56.2 ng/g lipid	27	25	1.36	0.57	3.25	trend 0.40	
			PCB 153	>56.2-71.3 ng/g lipid	16	25	0.80	0.32	2.03		
				>71.3 ng/g lipid	34	25	1.59	0.63	4.00		
				per 10 ng/g lipid	--	--	*1.03*	*0.96*	*1.12*		
				≤12.2 ng/g lipid	22	25	1.0	reference			
				>12.2-17 ng/g lipid	16	24	0.84	0.36	1.92	trend 0.13	Age, sex, study site, date of blood draw
			PCB 170	>17-22.5 ng/g lipid	27	24	1.59	0.63	4.02		
				>22.5 ng/g lipid	31	24	1.73	0.73	4.14		
				per 10 ng/g lipid	--	--	*1.24*	*0.94*	*1.63*		
				≤28.7 ng/g lipid	16	26	1.0	reference			
				>28.7-41.2 ng/g lipid	21	24	1.72	0.65	4.54	**trend 0.01**	
			PCB 180	>41.2-54.4 ng/g lipid	22	25	1.82	0.70	4.76		
				>54.4 ng/g lipid	41	25	**3.50**	**1.34**	**9.15**		
				per 10 ng/g lipid	--	--	*1.08*	*0.98*	*1.20*		
				≤2.7 ng/g lipid	30	25	1.0	reference			
				>2.7-4.4 ng/g lipid	21	20	0.93	0.16	5.46	trend 0.96	
			PCB 183	>4.4-6.3 ng/g lipid	22	27	0.73	0.26	2.06		
				>6.3 ng/g lipid	27	28	1.02	0.36	2.93		
				per 10 ng/g lipid	--	--	*0.91*	*0.42*	*1.97*		
				≤8.8 ng/g lipid	24	25	1.0	reference			
				>8.8-12.0 ng/g lipid	13	25	0.59	0.22	1.57	trend 0.18	
			PCB 187	>12.0-18.0 ng/g lipid	33	25	1.34	0.59	3.04		
				>18.0 ng/g lipid	30	25	1.22	0.49	3.08		
				per 10 ng/g lipid	--	--	*1.09*	*0.84*	*1.42*		
				≤7.9 ng/g lipid	17	25	1.0	reference			
				>7.9-11.2 ng/g lipid	24	25	1.59	0.62	4.04	**trend 0.04**	
			PCB 194	>11.2-15.6 ng/g lipid	20	25	1.35	0.53	3.48		
				>15.6 ng/g lipid	37	25	**2.68**	**1.04**	**6.90**		
				per 10 ng/g lipid	--	--	*1.45*	*0.98*	*2.15*		

Supplemental Material, Table S1 (continued).

Citation<br>Study Population	PCB source	Control Matching Variables	PCB Congener or Group	Exposure Level (lipid adjusted)	# Cases	# Controls	OR [a]	95% CI		p-value	Adjustment Variables [b]
**Engel et al, 2007**	Janus cohort: Serum	Janus cohort:	**Σ PCB**								
Janus,	CLUE I cohort: Serum	Sex, county, age at	Janus cohort	Median 1048.3 ng/g lipid	44	48	1.0	reference		NR	BMI, smoking status, sex, county, age at examination, date of examination
CLUE I,	NHS cohort: Plasma	examination, date of		Median 1398.3 ng/g lipid	48	47	1.1	0.7	2.0		
Nurses' Health Study		examination		Median 1674.9 ng/g lipid	38	48	1.0	0.5	1.9		
(NHS)		CLUE I cohort:		Median 2148.2 ng/g lipid	60	47	1.7	0.8	3.4		
		Race, sex, date of birth,	CLUE I cohort	Median 551.4 ng/g lipid	10	37	1.0	reference		**trend <0.005**	Education, current smoking status, race, sex, date of birth, date of blood sample
		date of blood sample		Median 726.0 ng/g lipid	13	37	1.6	0.6	4.3		
		donation		Median 911.5 ng/g lipid	21	37	**3.0**	**1.1**	**8.3**		
		NHS cohort:		Median 1377.3 ng/g lipid	30	36	**4.6**	**1.7**	**12.7**		
		None	NHS cohort	Median 672.6 ng/g lipid	3	26	1.0	reference		--	BMI, smoking status, age, p,p'-DDE
				Median 915.2 ng/g lipid	12	26	3.5	0.8	14.8		
				Median 1092.3 ng/g lipid	15	26	4.3	1.0	18.2		
			**PCB 118**								
			Janus cohort	Median 61.5 ng/g lipid	42	48	1.0	reference		**trend <0.05**	BMI, smoking status, sex, county, age at examination, date of examination
				Median 80.6 ng/g lipid	43	48	1.0	0.5	2.0		
				Median 100.0 ng/g lipid	47	48	1.2	0.6	2.3		
				Median 138.7 ng/g lipid	58	46	1.7	0.9	3.5		
			CLUE I cohort	Median 99.4 ng/g lipid	5	37	1.0	reference		**trend <0.05**	Education, current smoking status, race, sex, date of birth, date of blood sample
				Median 124.6 ng/g lipid	23	37	**4.9**	**1.6**	**15.3**		
				Median 164.9 ng/g lipid	17	37	3.5	1.0	11.8		
				Median 214.7 ng/g lipid	29	36	**5.4**	**1.7**	**17.1**		
			NHS cohort	Median 52.1 ng/g lipid	5	26	1.0	reference		--	BMI, smoking status, age, p,p'-DDE
				Median 74.8 ng/g lipid	13	26	3.5	0.9	13.0		
				Median 107.7 ng/g lipid	12	26	2.8	0.7	11.5		
			**PCB 138**								
			Janus cohort	Median 91.2 ng/g lipid	51	48	1.0	reference		**trend <0.05**	BMI, smoking status, sex, county, age at examination, date of examination
				Median 122.8 ng/g lipid	29	47	0.6	0.3	1.2		
				Median 153.4 ng/g lipid	42	48	0.9	0.5	1.7		
				Median 190.0 ng/g lipid	68	47	1.7	0.8	3.2		
			CLUE I cohort	Median 95.1 ng/g lipid	8	37	1.0	reference		**trend <0.05**	Education, current smoking status, race, sex, date of birth, date of blood sample
				Median 129.1 ng/g lipid	20	37	2.5	0.9	6.5		
				Median 164.5 ng/g lipid	19	37	2.7	1.0	7.5		
				Median 242.4 ng/g lipid	27	36	**4.4**	**1.5**	**12.6**		
			NHS cohort	Median 64.8 ng/g lipid	4	26	1.0	reference		--	BMI, smoking status, age, p,p'-DDE
				Median 104.9 ng/g lipid	10	26	2.3	0.6	9.4		
				Median 157.1 ng/g lipid	16	26	3.4	0.9	13.0		
			**PCB 153**								
			Janus cohort	Median 194.5 ng/g lipid	40	48	1.0	reference		**trend <0.05**	BMI, smoking status, sex, county, age at examination, date of examination
				Median 268.1 ng/g lipid	44	48	1.2	0.6	2.3		
				Median 330.2 ng/g lipid	43	47	1.2	0.7	2.2		
				Median 417.3 ng/g lipid	63	47	2.0	1.0	3.9		
			CLUE I cohort	Median 92.6 ng/g lipid	16	37	1.0	reference		**trend <0.05**	Education, current smoking status, race, sex, date of birth, date of blood sample
				Median 122.4 ng/g lipid	14	37	1.0	0.4	2.3		
				Median 163.2 ng/g lipid	17	37	1.4	0.5	3.5		
				Median 246.9 ng/g lipid	27	36	2.2	0.9	5.2		
			NHS cohort	Median 82.8 ng/g lipid	4	26	1.0	reference		--	BMI, smoking status, age, p,p'-DDE
				Median 120.3 ng/g lipid	13	26	2.7	0.7	10.0		
				Median 180.0 ng/g lipid	13	26	2.7	0.7	10.5		

Supplemental Material, Table S1 (continued).

Citation<br>Study Population	PCB source	Control Matching Variables	PCB Congener or Group	Exposure Level (lipid adjusted)	# Cases	# Controls	OR [a]	95% CI		p-value	Adjustment Variables [b]
**Fritschi et al, 2005**<br>NHL cases and population control residents of New South Wales or the Australian Capital Territory	Occupational exposure estimated by occupational hygienist based on self-reported occupational history	Frequency matched on age, sex, region of residence	Ever occupationally exposed to PCBs	No	682	681	1.0	reference		--	Age, sex, state of residence, ethnic origin
				Yes	12	13	1.10	0.49	2.44		
			Probability of exposure to PCBs	No	NR	NR	1.0	reference		--	
				Possible	NR	NR	0.40	0.12	1.31		
				Probable	NR	NR	4.54	0.97	21.2		
			Level of exposure to PCBs	None	NR	NR	1.0	reference		trend 0.90	
				Low	NR	NR	1.91	0.75	4.85		
				Medium	NR	NR	0.78	0.17	3.5		
				High	NR	NR	---	---	---		
			Frequency of exposure to PCBs	Never	NR	NR	1.0	reference		trend 0.60	
				≤4 days per year	NR	NR	1.44	0.49	4.22		
				>4 days per year	NR	NR	1.15	0.35	3.81		
			Years of exposure to PCBs	Never	NR	NR	1.0	reference		trend 0.80	
				<5 years	NR	NR	1.04	0.26	4.19		
				≥5 years	NR	NR	1.13	0.43	2.97		
**Greenland et al, 1994**<br>Mortality study among employees at a transformer manufacturing plant in the US	Occupational exposure estimated based on work history records	None.<br>Controls were workers who died of causes thought to be unrelated to the occupational exposures under study	Exposure to Pyranol (a mixture of ~50% PCBs and ~50% trichlorobenzene)	Indirect or no exposure	NR	NR	1.0	reference		trend >0.40	Age, death year
				Direct exposure	NR	NR	**3.26** [c]	**1.14**	**9.32**		
				Indirect or no exposure	NR	NR	1.0	reference		p=0.42	Age at death, death year, hire year
				97th percentile of exposure (in controls)	NR	NR	1.5 [c]	0.55	4.3		
**Hardell et al, 1996/1997**<br>NHL cases and surgical controls in Sweden	Adipose	Frequency matched on age and sex	Σ PCBs	below median (1300 ng/g lipid)	NR	NR	1.0	reference		--	--
				above median (1300 ng/g lipid)	16	NR	2.7	0.8	9.4		
			Σ PCBs	below median (1300 ng/g lipid)	NR	NR	1.0	reference		--	Age, sex
				above median (1300 ng/g lipid)	NR	NR	1.8	0.4	7.4		
**Hardell et al, 2001**<br>NHL cases, surgical controls, and population controls in Sweden	Adipose or plasma	Surgical patient controls (n=47) frequency matched on age and sex; Controls randomly sampled from population (n=36) matched on age and sex	Σ PCBs	below median (1020 ng/g lipid)	31	42	1.0	reference		--	Age, sex, BMI, specimen type
				above median (1020 ng/g lipid)	51	41	1.8	0.85	3.9		
			Σ PCBs	below median (1020 ng/g lipid)	53	51	1.0	reference		--	Age, sex, BMI, specimen type, hexachlorobenzene, p,p'-DDE, Σ chlordanes, TBDE
				above median (1020 ng/g lipid)	29	32	1.1	0.28	3.9		
			Σ Immunotoxic PCBs	below median (355 ng/g lipid)	25	40	1.0	reference		--	Age, sex, BMI, specimen type
				above median (355 ng/g lipid)	57	43	**3.2**	**1.4**	**7.4**		

Supplemental Material, Table S1 (continued).

**Citation** Study Population	**PCB source**	**Control Matching Variables**	**PCB Congener or Group**	**Exposure Level (lipid adjusted)**	**# Cases**	**# Controls**	**OR [a]**	**95% CI**		**p-value**	**Adjustment Variables [b]**
**Hardell et al, 2009** NHL cases and population controls in Sweden	Plasma	Frequency matched on age and sex	Σ PCBs	below median (646 ng/g lipid)	40	50	1.0	reference		--	Age, sex, BMI
				above median (646 ng/g lipid)	59	49	2.0	0.99	3.9		
			Σ Lower chlorinated PCBs	below median (8.1 ng/g lipid)	52	50	1.0	reference		--	
				above median (8.1 ng/g lipid)	47	49	1.1	0.6	2.0		
			Σ Moderately chlorinated PCBs	below median (559 ng/g lipid)	41	50	1.0	reference		--	
				above median (559 ng/g lipid)	58	49	1.8	0.9	3.6		
			Σ Higher chlorinated PCBs	below median (13 ng/g lipid)	36	50	1.0	reference		--	
				above median (13 ng/g lipid)	63	49	1.7	0.8	3.4		
			Σ Immunotoxic PCBs	below median (226 ng/g lipid)	45	50	1.0	reference		--	
				above median (226 ng/g lipid)	54	49	1.5	0.8	3.4		
**Laden et al, 2010** Nurses' Health Study	Plasma	Age, race, month of blood draw, fasting status at blood draw	Σ PCBs	median 406.9 ng/g lipid	33	72	1.0	reference		trend 0.76	Race, age at blood draw, year and month of blood draw, fasting status at blood draw, region, BMI, current smoking status, parity/ breastfeeding, height
				median 547.8 ng/g lipid	41	73	1.25	0.68	2.28		
				median 678.0 ng/g lipid	41	73	1.32	0.71	2.43		
				median 945.4 ng/g lipid	30	72	1.02	0.53	1.95		
			Σ Immunotoxic PCBs	median 75.6 ng/g lipid	34	72	1.0	reference		trend 0.48	
				median 111.5 ng/g lipid	56	73	**1.83**	**1.01**	**3.31**		
				median 149.6 ng/g lipid	30	73	0.94	0.51	1.76		
				median 228.7 ng/g lipid	25	72	0.89	0.45	1.77		
			Σ PCB 118, 138, 153, 180	median 185.7 ng/g lipid	33	72	1.0	reference		trend 0.63	
				median 257.5 ng/g lipid	36	73	1.04	0.57	1.92		
				median 334.4 ng/g lipid	48	73	1.63	0.90	2.95		
				median 471.7 ng/g lipid	28	72	0.91	0.48	1.75		
			PCB 118	median 27.4 ng/g lipid	38	72	1.0	reference		trend 0.42	
				median 42.9 ng/g lipid	49	73	1.39	0.78	2.47		
				median 61.0 ng/g lipid	31	73	0.89	0.48	1.64		
				median 104.7 ng/g lipid	27	72	0.81	0.42	1.56		
			PCB 138	median 34.3 ng/g lipid	31	72	1.0	reference		trend 0.59	
				median 53.2 ng/g lipid	39	73	1.33	0.73	2.40		
				median 75.7 ng/g lipid	48	73	1.61	0.89	2.92		
				median 113.3 ng/g lipid	27	72	0.95	0.49	1.83		
			PCB 153	median 64.9 ng/g lipid	37	72	1.0	reference		trend 0.55	
				median 91.2 ng/g lipid	33	73	0.85	0.47	1.54		
				median 120.3 ng/g lipid	45	73	1.38	0.76	2.51		
				median 170 ng/g lipid	30	72	0.82	0.43	1.56		
			PCB 180	median 47.8 ng/g lipid	36	72	1.0	reference		trend 0.82	
				median 63.4 ng/g lipid	33	73	1.02	0.54	1.93		
				median 80.5 ng/g lipid	44	73	1.24	0.66	2.31		
				median 109.4 ng/g lipid	32	72	1.03	0.52	2.02		

Supplemental Material, Table S1 (continued).

**Citation** Study Population	**PCB source**	**Control Matching Variables**	**PCB Congener or Group**	**Exposure Level (lipid adjusted)**	**# Cases**	**# Controls**	**OR** [a]	**95% CI**		**p-value**	**Adjustment Variables** [b]
**Maifredi et al, 2010** Residents of a municipality in Brescia, Italy with soil contamination by PCBs from a PCB-production factory	Lifetime residential history in a contaminated area (based on PCB concentrations in soil samples)<br><br>Area D: least contaminated area<br>Area A: most highly contaminated area<br>Area B: Intermediate levels of contamination<br>Area C: Intermediate levels of contamination (lower than area B)	Frequency matched on age and sex	Σ PCBs in soil *All cases (incident and deaths)*	area D	462	1394	1.0	reference			Age, gender
				area A	1	8	0.4	0.1	3.0	NR	
				area B	25	55	1.4	0.8	2.2	NR	
				area C	7	10	2.1	0.8	5.5	NR	
				areas A, B, C	33	73	1.4	0.9	2.1	NR	
			Σ PCBs in soil *Incident cases only*	area D	265	1394	1.0	reference			Age, gender
				area A	1	8	0.7	0.1	5.5	NR	
				area B	17	55	1.6	0.9	2.8	NR	
				area C	4	10	2.0	0.6	6.6	NR	
				areas A, B, C	22	73	1.6	0.9	2.5	p=0.08	
			Σ PCBs in soil *All cases (incident and deaths); 10+ years duration of residence*	area D	394	1208	1.0	reference			Age, gender
				area A	15	26	1.8	0.9	3.9	p=0.08	
				area B	55	129	1.3	0.9	1.8	NR	
				area C	12	25	1.5	0.7	3.0	NR	
				areas A, B, C	80	176	**1.4**	**1.1**	**1.8**	**p=0.02**	
			Σ PCBs in soil *Incident cases only; 10+ years duration of residence*	area D	394	1208	1.0	reference			Age, gender
				area A	8	26	1.7	0.7	3.8	NR	
				area B	31	129	1.2	0.8	1.9	NR	
				area C	6	25	1.2	0.5	3.0	NR	
				areas A, B, C	44	176	1.3	0.9	1.8	NR	
**Morton et al, 2008** NCI-SEER study	Carpet dust	Frequency matched on age, sex, race, study center	PCB 180	<20.8 ng/g	499	395	1.0	reference		trend 0.07	Age, sex, race, study center, education
				20.8-44.3 ng/g	92	60	1.3	0.9	1.9		
				≥44.4 ng/g	91	58	1.4	1.0	2.0		
**Nordstrom et al, 2000** Hairy-cell leukemia cases and population controls in Sweden	Plasma	Age, sex, county	Σ PCBs	≤831.6 ng/g lipid	31	27	1.0	reference		--	Age, BMI, exposure to herbicides, fungicides, insecticides, impregnating agents, organic solvents, animals, exhausts
				>831.6 ng/g lipid	23	27	0.8	0.3	1.9		
**Quintana et al, 2004** Cadaver and surgical patient adipose samples for NHL cases, accident victim controls, and MI controls from the US	Adipose	Age, sex, geographic region, race	Σ PCBs	Trace, not detected, or <1 ppm	79	184	1.0	reference		--	Year of sample collection
				1-3 ppm	50	151	1.05	0.63	1.76		
				>3 ppm	9	23	1.08	0.40	2.92		
**Rothman et al, 1997** CLUE I cohort	Serum	Race, sex, date of birth, participation in CLUE I or CLUE I/II, date of sample donation, participation in further surveys, location of specimen storage	Σ PCBs	247-641 ng/g lipid	10	37	1.00	reference		**trend 0.002**	DDT, race, sex, date of birth, participation in CLUE I or CLUE I/II, date of sample donation, participation in further surveys, location of specimen storage
				649-806 ng/g lipid	13	37	1.3	0.5	3.3		
				814-1060 ng/glipid	21	37	2.7	0.9	7.8		
				1070-2070 ng/g lipid	30	36	**4.1**	**1.4**	**11.9**		

Supplemental Material, Table S1 (continued).

**Citation** Study Population	**PCB source**	**Control Matching Variables**	**PCB Congener or Group**	**Exposure Level (lipid adjusted)**	**# Cases**	**# Controls**	**OR** [a]	**95% CI**		**p-value**	**Adjustment Variables** [b]
**Spinelli et al, 2007** NHL cases and population controls in British Columbia, Canada	Plasma	Frequency matched on sex, age, residential location	Σ PCBs	≤100.9 ng/g lipid	81	115	1.00	reference		**trend 0.001**	Age, family history of NHL
				>100.9-155.6 ng/g lipid	103	114	1.41	0.93	2.14		
				>155.6-220.0 ng/g lipid	77	115	1.11	0.71	1.74		
				>220.0-6571 ng/g lipid	142	115	**2.14**	**1.38**	**3.30**		
			Σ Dioxin-like PCBs	≤10.12 ng/g lipid	82	115	1.00	reference		**trend <0.001**	Age, farming
				>10.12-15.35 ng/g lipid	96	114	1.41	0.91	2.16		
				>15.35-23.72 ng/g lipid	82	115	1.57	1.00	2.46		
				>23.72-280.8 ng/g lipid	143	115	**2.40**	**1.53**	**3.77**		
			Σ Non-dioxin-like PCBs	≤88.57 ng/g lipid	85	115	1.00	reference		**trend <0.001**	Age, region, family history of NHL
				>88.57-136.2 ng/g lipid	96	115	1.30	0.85	1.97		
				>136.2-196.4 ng/g lipid	93	115	1.19	0.76	1.86		
				>196.4-6445 ng/g lipid	148	115	**2.18**	**1.41**	**3.38**		
			PCB 105	not detected	281	316	1.00	reference		trend 0.675	Education
				>1.32-37.65 ng/g lipid	132	139	1.06	0.80	1.42		
			PCB 118	≤4.57 ng/g lipid	82	109	1.00	reference		**trend 0.004**	Age, BMI, farming
				>4.57-7.78 ng/g lipid	88	113	1.12	0.74	1.69		
				>7.78-12.85 ng/g lipid	95	114	1.23	0.81	1.88		
				>12.85-202.1 ng/g lipid	129	113	**1.77**	**1.15**	**2.72**		
			PCB 156	≤3.65 ng/g lipid	85	114	1.00	reference		**trend 0.004**	Age, farming
				>3.65-5.51 ng/g lipid	85	114	1.10	0.72	1.68		
				>5.51-8.32 ng/g lipid	105	115	1.43	0.93	2.21		
				>8.32-113.3 ng/g lipid	128	115	**1.77**	**1.14**	**2.74**		
			PCB 28	not detected	348	376	1.00	reference		trend 0.779	None
				>1.38-54.47 ng/g lipid	74	84	0.95	0.67	1.34		
			PCB 99	≤3.06 ng/g lipid	106	113	1.00	reference		**trend 0.045**	Age, farming
				>3.06-4.83 ng/g lipid	82	115	0.78	0.52	1.15		
				>4.83-7.78 ng/g lipid	85	115	0.81	0.54	1.21		
				>7.78-61.34 ng/g lipid	130	115	1.27	0.86	1.87		
			PCB 138	≤11.61 ng/g lipid	100	115	1.00	reference		**trend 0.02**	Age
				>11.61-19.28 ng/g lipid	90	115	0.93	0.62	1.38		
				>19.28-29.72 ng/g lipid	94	115	0.99	0.66	1.50		
				>29.72-289.4 ng/g lipid	138	115	1.46	0.98	2.18		
			PCB 153	≤25.29 ng/g lipid	90	115	1.00	reference		**trend 0.002**	Age
				>25.29-38.68 ng/g lipid	86	115	1.04	0.68	1.57		
				>38.68-59.0 ng/g lipid	106	115	1.34	0.87	2.04		
				>59.0-735.9 ng/g lipid	140	115	**1.79**	**1.17**	**2.72**		
			PCB 170	≤7.16 ng/g lipid	88	115	1.00	reference		**trend 0.005**	Age
				>7.16-11.17 ng/g lipid	93	115	1.17	0.77	1.79		
				>11.17-17.23 ng/g lipid	107	115	1.41	0.91	2.18		
				>17.23-901.5 ng/g lipid	134	115	**1.80**	**1.16**	**2.79**		
			PCB 180	≤21.93 ng/g lipid	85	111	1.00	reference		**trend 0.005**	Age, sex, BMI, ethnicity, family history of NHL
				>21.93-35.63 ng/g lipid	94	110	1.28	0.82	2.00		
				>35.63-54.72 ng/g lipid	89	115	1.25	0.78	2.00		
				>54.72-3787 ng/g lipid	126	113	**1.91**	**1.19**	**3.07**		
			PCB 183	not detected	162	177	1.00	reference		trend 0.113	Age
				>1.87-3.95 ng/g lipid	107	142	0.83	0.59	1.18		
				>3.95-84.86 ng/g lipid	153	141	1.22	0.87	1.71		
			PCB 187	≤5.93 ng/g lipid	88	114	1.00	reference		**trend 0.003**	Age, education, family history of NHL
				>5.93-9.82 ng/g lipid	98	114	1.27	0.83	1.95		
				>9.82-15.46 ng/g lipid	79	114	1.04	0.66	1.63		
				>15.46-833.2 ng/g lipid	136	112	**1.92**	**1.23**	**2.98**		

**Citation** Study Population	**PCB source**	**Control Matching Variables**	**PCB Congener or Group**	**Exposure Level (lipid adjusted)**	**# Cases**	**# Controls**	**OR** [a]	**95% CI**		**p-value**	**Adjustment Variables** [b]
**Viel et al, 2011** Residents of an area of Besancon, France with high levels of dioxin emissions from a municipal solid waste incinerator	Serum	Sex, age, date of blood draw	Σ Dioxin-like PCBs	per pg WHO-TEQ/g lipid	--	--	1.04	1.00	1.07	**p=0.02**	None
			Σ Non-dioxin-like PCBs	per 10 ng/g lipid	--	--	**1.02**	**1.01**	**1.05**	**p=0.01**	
			Σ PCDD, PCDF, dioxin-like PCBs	per pg WHO-TEQ/g lipid	--	--	**1.04**	**1.01**	**1.05**	**p=0.01**	
			PCB 77	per pg WHO-TEQ/g lipid	--	--	∞	--	--	p=0.4	
			PCB 126	per pg WHO-TEQ/g lipid	--	--	1.10	1.00	1.23	**p=0.04**	
			PCB 169	per pg WHO-TEQ/g lipid	--	--	**3.40**	**1.11**	**13.04**	**p=0.03**	
			PCB 105	per pg WHO-TEQ/g lipid	--	--	**3.29**	**1.07**	**13.41**	**p=0.02**	
			PCB 114	per pg WHO-TEQ/g lipid	--	--	1.55	0.97	2.84	p=0.06	
			PCB 118	per pg WHO-TEQ/g lipid	--	--	**1.21**	**1.01**	**1.53**	**p=0.02**	
			PCB 123	per pg WHO-TEQ/g lipid	--	--	∞	--	--	**p=0.02**	
			PCB 156	per pg WHO-TEQ/g lipid	--	--	1.09	1.00	1.20	**p=0.02**	
			PCB 157	per pg WHO-TEQ/g lipid	--	--	**1.41**	**1.03**	**2.13**	**p=0.03**	
			PCB 167	per pg WHO-TEQ/g lipid	--	--	∞	--	--	**p=0.02**	
			PCB 189	per pg WHO-TEQ/g lipid	--	--	**85.4**	**2.12**	**7349.0**	**p=0.01**	
			PCB 138	per 10 ng/g lipid	--	--	**1.08**	**1.01**	**1.20**	**p=0.03**	
			PCB 153	per 10 ng/g lipid	--	--	1.04	1.00	1.09	**p=0.03**	
			PCB 180	per 10 ng/g lipid	--	--	**1.08**	**1.01**	**1.17**	**p=0.01**	

Abbreviations:

BMI, body mass index; **CI,** confidence interval; **CLUE I,** Campaign Against Cancer and Stroke cohort study; **DDE,** dichlorodiphenyldichloroethylene; **DDT,** dichlorodiphenyltrichloroethane; **MI,** myocardial infarction; ng/g, nanograms per gram; **NHL,** non-Hodgkin lymphoma; **NHS,** Nurses' Health Study; **NR,** not reported; **OR,** odds ratio; **PCB,** polychlorinated biphenyl; pg/g, picograms per gram; **ppb,** parts per billion; **ppm,** parts per million; **RR,** relative risk; **TBDE,** 2,2',4,2'-tetrabrominated diphenyl ether; **TEQ,** toxic equivalency quotient; **UDL,** usual detection limit; **WHO,** World Health Organization

[a] Results presented only for main effects of PCB exposure on NHL (overall). NHL subtype-specific analyses not presented.

[b] In studies with multiple models, fully adjusted models are shown.

[c] Odds of lymphoma death.

bold face: statistically significant results at an alpha of 0.05

Supplemental Material, Table S2. Characteristics of Cohort Studies Examining the Association Between Occupational PCB Exposure and NHL or Lymphoma

Citation	Cohort	Cohort Size (n)	Deaths (n)	Duration of Follow-Up	Notes on Cohort Age or Exposure	Outcome	O/E	SMR/SIR (95% CI)
Brown and Jones 1981	Capacitor manufacturers (NY, MA)	2,567	163	35 y (1940-1975)	≥30% of cohort employed for ≤2 y	*Mortality*: L & H	2 / 4.34	0.46 (p>0.05)
Brown 1987	Capacitor manufacturers (NY, MA)	2,588	295	42 y (1940-1982)	See Brown and Jones 1981	*Mortality*: L & H	5 / 7.40	0.68 (p>0.05)
Prince et al. 2006a	Capacitor manufacturers (NY, MA)	2,572	798	58 y (1940-1998)	Median age at first employment= 23.3 y; 49% of cohort employed <5 y	*Mortality*: NHL	10 / 7.63	1.31 (0.63,2.41)
Prince et al. 2006b	Capacitor manufacturers (NY, MA)	14,458	3,417	58 y (1940-1998)	75% of cohort left employment before age 55	*Mortality*: NHL	35 / 35.71	0.98 (0.68,1.36)
Kimbrough et al. 1999	Capacitor manufacturers (NY)	7,075	1,195	31 y (1946-1977)	Mean duration of employment= 5.6 y; Mean age at start of employment= 27 y	*Mortality*: LS &RS	3 / 4.62	0.65 (0.13,1.90)
						Mortality: Other L	14 / 14.2	0.98 (0.54,1.65)
Kimbrough et al. 2003	Capacitor manufacturers (NY)	7,075	1,654	52 y (1946-1998)	See Kimbrough et al. 1999	*Mortality*: LS &RS	3 / 4.8	0.63 (0.13,1.83)
						Mortality: Other L	22 / 21.2	1.04 (0.65,1.57)
Sinks et al. 1992	Capacitor manufacturers (IN)	3,588	192	19 y (1957-1986)	NR	*Mortality*: L & H	7 / 7.20	1.00 (0.40,2.00)
Ruder et al. 2006	Capacitor manufacturers (IN)	3,569	547	38 y (1960-1998)	Median age at first employment= 24 y; Median duration of employment=1.3 y	*Mortality*: NHL	9 / 7.32	1.23 (0.60,2.30)

Supplemental Material, Table S2 (continued).

Citation	Cohort	Cohort Size (n)	Deaths (n)	Duration of Follow-Up	Notes on Cohort Age or Exposure	Outcome	O/E	SMR/SIR (95% CI)
Bertazzi et al. 1982	Capacitor manufacturers (Italy)	1,310	27	32 y (1946-1978)	Employment ≥ 6 months required for inclusion; Authors noted “very young age” of workers	*Mortality*: L & H	4 / 0.91	4.4 (1.2,12.2)
Bertazzi et al. 1987	Capacitor manufacturers (Italy)	2,100	30	36 y (1946-1982)	NR	*Mortality*: Hemato	3 / 0.80	3.75 (p>0.05)
Tironi et al. 1996	Capacitor manufacturers (Italy)	1,556	47	36 y (1946-1982)	NR	*Mortality*: L & H	5 / 3.50	1.41 (0.46,3.30)
Mallin et al. 2004	Capacitor manufacturers (IL)	2,885	1,199	66 y (1944-2000)	>55% of cohort employed for ≤1 y	*Mortality*: LS & RS	3 / 1.96	1.53 (0.32,4.48)
						Mortality: NHL [a]	13 / 7.39	1.76 (0.94,3.02)
Gustavsson et al. 1997	Capacitor manufacturers (Sweden)	241	56	26 y (1965-1991)	NR	*Mortality*: Lymphoma	1 / 0.39	2.54 (0.07,14.2)
			18 incident cancers			*Incidence*: NHL	1 / 0.67	1.49 (0.02,8.30)
Liss et al. 1989 [b]	Transformer manufacturers (Ontario)	1,073	146	25 y (1960-1985)	N/A	*Mortality*: L & H	2 / 0.69	2.90 (0.3,12.4)
Yassi et al. 1994	Transformer manufacturers (Canada)	812	71	39 y (1950-1989)	NR	*Mortality*: NHL	2 / 0.79	2.54 (0.29,9.17)
Loomis et al. 1997	Electrical power company workers (U.S.)	138,905	20,733	38 y (1950-1988)	Authors noted cohort was “relatively young”	*Mortality*: LS & RS	69 / 89.6	0.77 (0.60,0.97)
						Mortality: Other L [c]	176 / 169.9	1.04 (0.89,1.20)

Supplemental Material, Table S2 (continued).

Citation	Cohort	Cohort Size (n)	Deaths (n)	Duration of Follow-Up	Notes on Cohort Age or Exposure	Outcome	O/E	SMR/SIR (95% CI)
Svensson et al. 1995	Fishermen from the east coast of Sweden with historically high intake of fatty fish containing POPs	2,896	421	54 y (1935-1988)	Inclusion required membership in fishermen's organization for ≥1 year	*Mortality*: NHL	3 / 2.5	1.20 (0.25-3.49)
			197 incident cancers	20 y (1968-1988)		*Incidence*: NHL	5 / 5.8	0.85 (0.28-1.99)

Abbreviations: **CI,** confidence interval; **E**, expected number of cases in overall cohort; **Hemato**, hematologic malignancies (ICD-8 200-209); **IL**, Illinois; **IN**, Indiana; **L & H**, lymphatic and hematopoietic malignancies; **LS & RS**, lymphosarcoma and reticulosarcoma (ICD-9 200); **MA**, Massachusetts; **N/A**, not available; **NHL**, non-Hodgkin lymphoma; **NR**, no information reported by the authors on age or exposure duration characteristics of the cohort; **NY**, New York; **O**, observed number of cases in overall cohort; **Other L**, other lymphatic and hematopoietic malignancies (ICD-9 202-203); **POPs**, persistent organic pollutants; **SIR**, standardized incidence ratio; **SMR**, standardized mortality ratio; **y**, years.

[a] Among white females in an analysis restricted to 1960-1999, during which time it was possible to re-code malignancies as NHL (data for males not reported).
[b] Data obtained from Ruder et al., 2006.
[c] Includes ICD-9 202 only.

Supplemental Material, Table S3. Interaction Between PCBs and EBV and Risk of NHL

Citation	PCB Congener Grouping	Exposure Level [a]	Cases (n)	Controls (n)	OR (95% CI) [b]	Cases (n)	Controls (n)	OR (95% CI) [b]
Rothman et al. 1997				**EBV-EA <20**			**EBV-EA ≥20**	
	Σ PCBs	<810 ng/g lipid	18	62	1.0 (reference)	4	11	1.0 (0.3, 3.6)
		≥810 ng/g lipid	39	67	2.8 (1.2, 6.2)	12	3	22.3 (4.3, 115.0)
Hardell et al. 2001				**EBV-EA ≤80**			**EBV-EA >80**	
	Σ PCBs	≤1,018 ng/g lipid	10	15	1.0 (reference)	16	22	1.1 (0.39, 3.4)
		>1,018 ng/g lipid	17	25	1.6 (0.52, 5.1)	22	12	4.0 (1.2, 14.0)
	Σ Immunotoxic PCBs [c]	≤348 ng/g lipid	9	18	1.0 (reference)	13	19	1.4 (0.47, 4.3)
		>348 ng/g lipid	18	22	3.2 (0.99, 11.0)	25	15	6.4 (1.9, 2.4)
Hardell et al. 2009				**EBV-EA ≤40**			**EBV-EA >40**	
	Σ PCBs	≤646 ng/g lipid	14	25	1.0 (reference)	24	24	2.5 (0.97, 6.4)
		>646 ng/g lipid	20	29	2.1 (0.7, 6.1)	39	20	5.2 (1.9, 14.0)
	Σ Lower chlorinated PCBs [c]	≤8.1 ng/g lipid	16	26	1.0 (reference)	34	23	3.1 (1.3, 7.4)
		>8.1 ng/g lipid	18	28	1.6 (0.6, 4.2)	29	21	3.0 (1.2, 7.8) [d]
	Σ Moderately chlorinated PCBs [c]	≤559 ng/g lipid	14	25	1.0 (reference)	25	24	2.5 (0.99, 6.5)
		>559 ng/g lipid	20	29	2.0 (0.7, 5.8)	38	20	5.0 (1.8, 14.0)
	Σ Higher chlorinated PCBs [c]	≤13 ng/g lipid	14	23	1.0 (reference)	20	26	1.5 (0.6, 3.8)
		>13 ng/g lipid	20	31	1.1 (0.4, 3.0)	43	18	3.9 (1.4, 10.0)
	Σ Immunotoxic PCBs [c]	≤226 ng/g lipid	17	24	1.0 (reference)	26	25	1.9 (0.8, 4.6)
		>226 ng/g lipid	17	30	1.2 (0.4, 3.4)	37	19	3.7 (1.4, 9.6)
Nordstrom et al. 2000 [e]				**EBV-EA <40**			**EBV-EA ≥40**	
	Σ PCBs	≤831.6 ng/g lipid	15	23	1.0 (reference)	16	4	4.4 (0.99, 23.4)
		>831.6 ng/g lipid	10	21	0.4 (0.1, 1.4)	13	6	4.4 (1.2, 18.5)
	Σ Immunotoxic PCBs [c]	≤285.4 ng/g lipid	14	20	1.0 (reference)	14	7	1.7 (0.4, 7.3)
		>285.4 ng/g lipid	11	24	0.4 (0.1, 1.5)	15	3	11.3 (2.3, 73.1)

Abbreviations: **CI**, confidence interval; **EBV-EA**, Epstein-Barr Virus Early Antigen Diffuse and Restricted components (R+D); **ng/g**, nanograms per gram; **NHL**, non-Hodgkin lymphoma; **OR**, odds ratio; **PCB**, polychlorinated biphenyl.

[a] Dichotomized at the median level in controls. [b] Adjusted odds ratios. [c] As defined by Moysich et al. (1999) [d] Less than expected for biologic interaction [e] Hairy cell leukemia, a subtype of NHL.

Supplemental Material, References.

Bertazzi PA, Riboldi L, Pesatori AC, Radice L, Zocchetti C. 1987. Cancer mortality of capacitor manufacturing workers. Am J Ind Med 11:165-176.

Bertazzi PA, Zocchetti C, Guercilena S, Della Foglia M, Pesatori AC, Riboldi L. 1982. Mortality study of male and female workers exposed to PCB's. In: Prevention of occupational cancer - International Symposium Geneva: International Labour Office.

Bertrand KA, Spiegelman D, Aster JC, Altshul LM, Korrick SA, Rodig SJ, et al. 2010. Plasma organochlorine levels and risk of non-Hodgkin lymphoma in a cohort of men. Epidemiology 21:172-180.

Brown DP. 1987. Mortality of workers exposed to polychlorinated biphenyls - an update. Arch Environ Health 42:333-339.

Brown DP, Jones M. 1981. Mortality and industrial hygiene study of workers exposed to polychlorinated biphenyls. Arch Environ Health 36:120-129.

Cocco P, Brennan P, Ibba A, de Sanjose LS, Maynadie M, Nieters A, et al. 2008. Plasma polychlorobiphenyl and organochlorine pesticide level and risk of major lymphoma subtypes. Occup Environ Med 65:132-140.

Colt JS, Rothman N, Severson RK, Hartge P, Cerhan JR, Chatterjee N, et al. 2009. Organochlorine exposure, immune gene variation, and risk of non-Hodgkin lymphoma. Blood 113:1899-1905.

Colt JS, Severson RK, Lubin J, Rothman N, Camann D, Davis S, et al. 2005. Organochlorines in carpet dust and non-Hodgkin lymphoma. Epidemiology 16:516-525.

De Roos AJ, Hartge P, Lubin JH, Colt JS, Davis S, Cerhan JR, et al. 2005. Persistent organochlorine chemicals in plasma and risk of non-Hodgkin's lymphoma. Cancer Res 65:11214-11226.

Engel LS, Laden F, Andersen A, Strickland PT, Blair A, Needham LL, et al. 2007a. Polychlorinated biphenyl levels in peripheral blood and non-Hodgkin's lymphoma: a report from three cohorts. Cancer Res 67:5545-5552.

Fritschi L, Benke G, Hughes AM, Kricker A, Vajdic CM, Grulich A, et al. 2005. Risk of non-Hodgkin lymphoma associated with occupational exposure to solvents, metals, organic dusts and PCBs (Australia). Cancer Causes Control 16:599-607.

Greenland S, Salvan A, Wegman DH, Hallock MF, Smith TJ. 1994. A case-control study of cancer mortality at a transformer-assembly facility. Int Arch Occup Environ Health 66:49-54.

Gustavsson P, Hogstedt C. 1997. A cohort study of Swedish capacitor manufacturing workers exposed to polychlorinated biphenyls (PCBs). Am J Ind Med 32:234-249.

Hardell K, Carlberg M, Hardell L, Bjornfoth H, Jogsten IE, Eriksson M, et al. 2009. Concentrations of organohalogen compounds and titers of antibodies to Epstein-Barr virus antigens and the risk for non-Hodgkin lymphoma. Oncol Rep 21:1567-1576.

Hardell L, Eriksson M, Lindstrom G, Van BB, Linde A, Carlberg M, et al. 2001. Case-control study on concentrations of organohalogen compounds and titers of antibodies to Epstein-Barr virus antigens in the etiology of non-Hodgkin lymphoma. Leuk Lymphoma 42:619-629.

Hardell L, Liljegren G, Lindstrom G, Van BB, Fredrikson M, Hagberg H. 1997. Polychlorinated biphenyls, chlordanes, and the etiology of non-Hodgkin's lymphoma. Epidemiology 8:689.

Hardell L, van Bavel B, Lindstrom G, Fredrikson M, Hagberg H, Liljegren G, et al. 1996. Higher concentrations of specific polychlorinated biphenyl congeners in adipose tissue from non-Hodgkin's lymphoma patients compared to controls without malignant disease. Int J Oncol 9:603-608.

Kimbrough RD, Doemland ML, LeVois ME. 1999. Mortality in male and female capacitor workers exposed to polychlorinated biphenyls. J Occup Environ Med 41:161-171.

Kimbrough RD, Doemland ML, Mandel JS. 2003. A mortality update of male and female capacitor workers exposed to polychlorinated biphenyls. J Occup Environ Med 45:271-282.

Laden F, Bertrand KA, Altshul LM, Aster JC, Korrick SA, Sagiv SK. 2010. Plasma organochlorine levels and risk of non-Hodgkin lymphoma in the Nurses' Health Study. Cancer Epidemiol Biomarkers Prev 19:1381-1384.

Liss GM. 1989. Mortality and cancer morbidity among transformer manufacturing workers. Toronto:Ontario Ministry of Labour Policy and Regulations Branch Health Studies Service.

Loomis D, Browning SR, Schenck AP, Gregory E, Savitz DA. 1997. Cancer mortality among electric utility workers exposed to polychlorinated biphenyls. Occup Environ Med 54:720-728.

Mallin K, McCann K, D'Aloisio A, Freels S, Piorkowski J, Dimos J, et al. 2004. Cohort mortality study of capacitor manufacturing workers, 1944-2000. J Occup Environ Med 46:565-576.

Morton LM, Wang SS, Cozen W, Linet MS, Chatterjee N, Davis S, et al. 2008. Etiologic heterogeneity among non-Hodgkin lymphoma subtypes. Blood 112:5150-5160.

Moysich KB, Mendola P, Schisterman EF, Freudenheim JL, Ambrosone CB, Vena JE, et al. 1999. An evaluation of proposed frameworks for grouping polychlorinated biphenyl (PCB) congener data into meaningful analytic units. Am J Ind Med 35:223-231.

Nordstrom M, Hardell L, Lindstrom G, Wingfors H, Hardell K, Linde A. 2000. Concentrations of organochlorines related to titers to Epstein-Barr virus early antigen IgG as risk factors for hairy cell leukemia. Environ Health Perspect 108:441-445.

Prince MM, Hein MJ, Ruder AM, Waters MA, Laber PA, Whelan EA. 2006a. Update: cohort mortality study of workers highly exposed to polychlorinated biphenyls (PCBs) during the manufacture of electrical capacitors, 1940-1998. Environ Health 5:13. doi: 10.1186/1476-069X-5-13.

Prince MM, Ruder AM, Hein MJ, Waters MA, Whelan EA, Nilsen N, et al. 2006b. Mortality and exposure response among 14,458 electrical capacitor manufacturing workers exposed to polychlorinated biphenyls (PCBs). Environ Health Perspect 114:1508-1514.

Quintana PJE, Delfino RF, Korrick S, Ziogas A, Kutz FW, Jones EL, et al. 2004. Adipose tissue levels of organochlorine pesticides and polychlorinated biphenyls and risk of non-Hodgkin's lymphoma. Environ Health Perspect 112:854-861.

Rothman N, Cantor KP, Blair A, Bush D, Brock JW, Helzlsouer K, et al. 1997. A nested case-control study of non-Hodgkin lymphoma and serum organochlorine residues. Lancet 350:240-244.

Ruder AM, Hein MJ, Nilsen N, Waters MA, Laber P, Davis-King K, et al. 2006. Mortality among workers exposed to polychlorinated biphenyls (PCBs) in an electrical capacitor manufacturing plant in Indiana: an update. Environ Health Perspect 114:18-23.

Sinks T, Steele G, Smith AB, Watkins K, Shults RA. 1992. Mortality among workers exposed to polychlorinated biphenyls. Am J Epidemiol 136:389-398.

Spinelli JJ, Ng CH, Weber JP, Connors JM, Gascoyne RD, Lai AS, et al. 2007. Organochlorines and risk of non-Hodgkin lymphoma. Int J Cancer 121:2767-2775.

Svensson BG, Mikoczy Z, Stromberg U, Hagmar L. 1995. Mortality and cancer incidence among Swedish fishermen with a high dietary intake of persistent organochlorine compounds. Scand J Work Environ Health 21:106-115.

Tironi A, Pesatori AC, Consonni D, Zocchetti C, Bertazzi PA. 1996. Mortalita di lavoratrici esposte a PCB [The mortality of female workers exposed to PCBs] (Italian). Epidemiol Prev 20:200-202.

Viel JF, Floret N, Deconinck E, Focant JF, De Pauw E, Cahn JY. 2011. Increased risk of non-Hodgkin lymphoma and serum organochlorine concentrations among neighbors of a municipal solid waste incinerator. Environ Int 37:449-453.

Yassi A, Tate R, Fish D. 1994. Cancer mortality in workers employed at a transformer manufacturing plant. Am J Ind Med 25:425-437.
